# Who consumes ultra-processed food? A systematic review of sociodemographic determinants of ultra-processed food consumption from nationally representative samples

**DOI:** 10.1017/S0954422423000240

**Published:** 2023-10-31

**Authors:** Samuel J. Dicken, Sulmaaz Qamar, Rachel L. Batterham

**Affiliations:** 1 Centre for Obesity Research, Department of Medicine, University College London (UCL), London WC1E 6JF, UK; 2 Bariatric Centre for Weight Management and Metabolic Surgery, University College London Hospital (UCLH), London NW1 2BU, UK; 3 National Institute for Health Research, Biomedical Research Centre, University College London Hospital (UCLH), London W1T 7DN, UK

**Keywords:** ultra-processed, diet, sociodemographic determinants, health inequality

## Abstract

Ultra-processed food (UPF) intake is associated with increased non-communicable disease risks. However, systematic reports on sociodemographic predictors of UPF intake are lacking. This review aimed to understand UPF consumption based on sociodemographic factors, using nationally representative cohorts. The systematic review was pre-registered (PROSPERO:CRD42022360199), following PRISMA guidelines. PubMed/MEDLINE searches (‘ultra-processed/ultraprocessed’ and ‘ultra-processing/ultraprocessing’) until 07/09/2022 retrieved 1,131 results. Inclusion criteria included: observational, nationally representative adult samples, in English, in peer-reviewed journals, assessing the association between sociodemographics and individual-level UPF intake defined by the NOVA classification. Exclusion criteria included: not nationally representative, no assessment of sociodemographics and individual-level UPF intake defined by NOVA. Risk of bias was assessed using the Newcastle-Ottawa Scale (NOS). 55 papers were included, spanning 32 countries. All 13 sociodemographic variables identified were significantly associated with UPF intake in ≥1 studies. Significant differences in UPF intake were seen across age, race/ethnicity, rural/urbanisation, food insecurity, income and region, with up to 10-20% differences in UPF intake (% total energy). Higher UPF intakes were associated with younger age, urbanisation, and being unmarried, single, separated or divorced. Education, income and socioeconomic status showed varying associations, depending on country. Multivariate analyses indicated that associations were independent of other sociodemographics. Household status and gender were generally not associated with UPF intake. NOS averaged 5.7/10. Several characteristics are independently associated with high UPF intake, indicating large sociodemographic variation in non-communicable disease risk. These findings highlight significant public health inequalities associated with UPF intake, and the urgent need for policy action to minimise social injustice-related health inequalities.

## Introduction

The global prevalence of obesity and adiposity-related non-communicable diseases has greatly risen in recent decades, with 2 billion adults now living with overweight or obesity ^(1)^, compared with just over 100 million in 1975 ^(2)^. An increasing concern in relation to rising levels of obesity-related disease has been the nutrition transition during the same time period, away from minimally processed foods (MPF), and towards greater consumption of ultra-processed foods (UPF) ^(3)^. The most commonly used food processing classification, but not necessarily the most validated, is the NOVA classification (not an acronym). As defined by NOVA as foods made using extracts and components of whole foods, UPFs typically contain five or more ingredients, produced using industrial methods and ingredients not used in the home, such as bulking agents, emulsifiers and additives ^(4)^. The nutrition transition and rising sales of UPFs has been driven by economic development, with urbanisation, shifts in workplace dynamics, expansion of multinational corporations, and with rising income levels at the national and individual level ^(3,5)^. As such, UPF sales are increasing around the world, with sales in middle-income countries (MIC) rapidly rising to the levels sold in high-income countries (HIC) ^(6,7)^.

Higher intakes of UPF are associated with a range of adverse health outcomes including obesity, type 2 diabetes, cardiometabolic disease and all-cause mortality ^(8,9)^. As a result, health organisations and national dietary guidelines including the American Heart Association and Brazilian Dietary Guidelines recommend limiting UPF intake ^(10,11)^, and calls have been made for systemic, national and international-level change to support individuals to reduce their UPF intake ^(12)^. However, to understand the effectiveness of universal strategies to reduce UPF intake, or whether regional policy action or targeted UPF interventions may differentially benefit certain sociodemographic groups, it is crucial to determine which individuals are consuming large quantities of UPF, and therefore who is at greatest risk of future health problems.

Previous reviews and studies suggest that higher UPF intakes are associated with male gender, younger age, lower education level and higher incomes ^(3,5,13–15)^. However, previous studies are subject to a number of methodological limitations. National food balance sheets provide a minimalist tool to understand national-level trends in food consumption ^(16)^, but preclude the ability to consider sociodemographic differences in food intake. Household Budget and Expenditure Surveys based on food purchases can be used as a proxy to estimate household or individual intakes and allow for sociodemographic stratification, but still include biases such as food waste and food loss, and preclude the ability to determine food consumption by specific individuals in the household ^(16)^. Dietary assessment using food consumption tools (e.g. 24-hour dietary recalls, food diaries and food frequency questionnaires (FFQ)) are considered optimal measures to assess individual-level food consumption ^(16)^.

Many of the large-scale, health-based cohort studies that assess individual-level UPF intake and their relationship with health outcomes also consider sociodemographic associations with UPF consumption ^(8)^. Such studies use convenience sampling methods that may include biases such as a healthy volunteer selection bias, or lead to underrepresentation or exclusion of sociodemographic strata, resulting in such samples not being generalisable to the national population. Smaller samples from cities or specific regions may be representative of such regions, but are also unlikely to accurately represent the national population. In combination with food consumption assessments, nationally representative samples typically obtained with complex, multistage probabilistic sampling are needed to provide country-level distributions of UPF intake by sociodemographic factors.

No study has systematically reviewed and formally synthesised papers assessing the association between sociodemographic factors and individual-level UPF intake from nationally representative samples. Current reviews of sociodemographic predictors of individual-level UPF intake are not systematic ^(13,14)^, and do not focus on studies that are nationally representative ^(13)^, or consider only age and gender ^(15)^ or systematically review nationally representative samples without considering sociodemographic predictors of UPF intake ^(17)^.

The objective of this review was to systematically synthesise the evidence regarding sociodemographic characteristics associated with a high, individual-level UPF intake as defined by NOVA, in nationally representative samples.

## Methods

This systematic review was pre-registered on PROSPERO (CRD42022360199), and conducted according to Preferred Reporting Items for Systematic Reviews Meta-Analysis (PRISMA) guidelines ^(18)^. The checklist is provided in the supplementary materials. The protocol was unchanged from pre-registration.

### Eligibility criteria

Eligibility criteria were considered based on the population, exposure, comparator, outcome (PECO) approach: population: nationally representative sample of adults (with or without children) from any country; exposure: any sociodemographic measure; comparator: other levels or strata of a sociodemographic measure; outcome: absolute or relative individual-level consumption of UPF, defined by the NOVA classification.

Studies were included if they were: written in English; an original article published in a peer-reviewed journal; from any date; a cross-sectional or longitudinal observational study; a nationally representative sample of adults (with or without children) from any country; statistically assessing the association between sociodemographic characteristics and UPF intake; with individual-level, relative or absolute UPF intake analysis (e.g. grams/day, %kcal/day). defined by the NOVA classification ^(4)^, using detailed food consumption assessment tools.

Papers were excluded if they: were not in English; were not published in a peer-reviewed journal; were a review, ecological study, interventional trial, laboratory study or animal study; were not a nationally representative sample of adults or a subgroup of a nationally representative sample (e.g. elderly adults only or females only); did not provide statistical measures of the association between sociodemographic variables and UPF intake; did not define UPF intake using the NOVA classification (or used NOVA, but the dietary outcome was determined using principal component or latent class analysis); only measured a subgroup of UPFs (e.g. only sugar-sweetened beverages, only sweet UPFs); did not directly assess individual-level dietary intake (e.g. UPF intake based on sales, household purchases).

### Search strategy and study selection

Identification of papers was achieved by searching Pubmed/MEDLINE until 07/09/2022. No filters or limits were placed on searches. Pubmed/MEDLINE was searched with the terms: “ultra-processed” OR “ultraprocessed” OR “ultra-processing” OR “ultraprocessing”, producing 1,131 results. Results were obtained and imported into Excel by SJD.

The selection process was independently conducted by two authors (SJD and SQ). To select papers, the authors developed a flowchart for full-text selection from title and abstract review, and a flowchart for full-text review to determine inclusion into the systematic review (provided in the supplementary materials). Flowcharts were based on the inclusion/exclusion criteria. Titles and abstracts were independently screened by both authors for relevance using the flowcharts. Full papers were then retrieved for eligibility analysis. All papers were manually screened, with no automation tools. After full-text screening, both authors met to discuss any disagreements. SJD then screened the references and citations of included papers, which were then agreed upon by two authors (SJD and SQ). As the selection process was predetermined prior to data extraction, all papers from the same nationally representative survey were considered for inclusion, as different papers from the same country and survey may report different sociodemographic predictors and/or cover different time periods. If papers were deemed eligible for the systematic review based on inclusion/exclusion criteria but contained no statistical assessment of UPF intake across sociodemographic variables (e.g. confidence intervals of mean intake across strata, p-values of proportions across quantiles of UPF intake), the authors were contacted to provide statistical detail.

Outcomes included absolute or relative (to total energy or food intake by weight) individual-level UPF intake (such as servings/day, kcal/day, percent (%) kcal/day or grams/day). Studies needed to report at least one unit of measurement for inclusion. The effect measures were the statistical assessments of an association between sociodemographic variables and UPF intake (e.g., for inferential statistics: beta coefficients or odds ratios and confidence intervals and/or p-values, for descriptive statistics: mean intakes and/or intakes across quantiles and confidence intervals and/or p-values, correlations and p-values).

### Data extraction

Two authors (SJD and SQ) independently and manually extracted data using a pre-specified template. The data extracted by the authors included: title; authors; date of publication; country; cohort; sampling method; analytical sample size; dietary assessment method; sociodemographic assessment method; primary measure of UPF intake; average UPF intake for the sample; sociodemographic variables; unadjusted or adjusted measures of association; UPF intake across sociodemographic strata and/or statistical measures of association.

### Assessment of methodological quality

Risk of bias was assessed using the Newcastle-Ottawa Scale (NOS) method adapted for cross-sectional observational studies (supplementary materials). Risk of bias was independently conducted by two authors (SJD and SQ), with disagreements resolved through discussion. No formal assessments were conducted for risk of reporting bias due to missing results.

A certainty assessment was conducted by considering the risk of bias scores, limitations in included studies, and gaps in the narrative analysis across each grouping (number of countries, range of sociodemographic predictors, range of multivariate analyses).

### Data Synthesis

Papers were presented in tabular format, reporting the key characteristics of each study, including risk of bias. No data conversion or handling of missing data was conducted prior to data presentation. Results are presented as a narrative synthesis due to the varied reporting methods and statistics across studies. Results are reported in terms of the number of distinct countries, number of distinct surveys and number of unique analyses. Papers were grouped for narrative analysis according to study characteristics from the variables extracted during data collection to assess heterogeneity of associations: by country, across countries, by sociodemographic variable, by country-level income, and by multivariate analyses. Simple descriptive fractions are provided of which predictors were significantly associated with UPF intake, and which were not. No sensitivity analyses were planned. For papers from the same survey, during the same years and with the same predictors, the multivariate analyses were prioritised in the narrative review.

## Results

### Study selection

**Figure 1:**
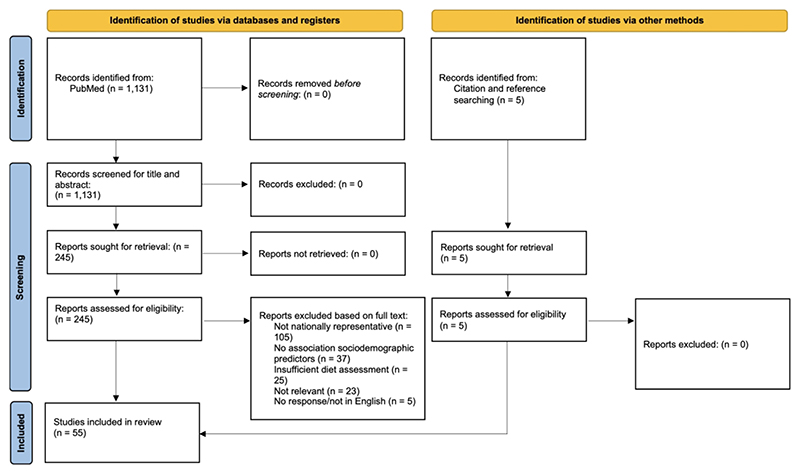
PRISMA Flow diagram of the systematic search process and study selection.

The database search retrieved 1,131 results. 245 results were retained for full-text screening. 55 studies were included in the systematic review. The results are detailed in the PRISMA flowchart (Figure 1).

Several studies appeared to meet the inclusion criteria, but were excluded as: authors did not respond to emails requesting further detail on statistical associations ^(19,20)^, statistical values were not available after request ^(21)^, or were not nationally representative ^(22,23)^. One study was excluded, as despite being a nationally representative cohort the authors explicitly outlined that the analytical sample could not be considered nationally representative ^(24)^. One case-control study was not deemed to be nationally representative, although the authors suggested it was ^(25)^. One paper from Brazil was excluded based on the use of a crude FFQ, reporting the frequency of consumption of 10 items to determine UPF intake ^(26)^. One paper from Mexico was included as participants were randomly selected from a nationally representative cohort, despite the small sample size ^(27)^.

### Study characteristics

In total, 55 studies were included in the systematic review, covering 32 countries and 36 nationally representative surveys. 54 studies were from 17 individual countries from 19 nationally representative surveys, with one additional study spanning 22 European countries ^(27–81)^. The main study characteristics are reported in Table 1. Sample size varied from 359 ^(32)^, to 57,423 ^(50)^. Five of the studies were included after considering citations of the included papers from the systematic search ^(28,35,39,61,63)^.

13 sociodemographic characteristics were assessed as predictors of UPF, including age, gender, race/ethnicity, income, education level, socioeconomic status/occupation/occupational social class, food security, marital status, household status (number and type of individuals (child, adult, elderly), rural/urban location, region of the country, immigrant status/country of birth and indigenous identity. One sociodemographic association was reported in 15 countries, and at least three sociodemographic associations were reported in 17 countries. Seven or more sociodemographic associations were reported in eight countries.

The included studies are detailed in Table 1, with statistical associations between sociodemographic variables and UPF intake reported in each study presented in Table 2.

### Associations by country

#### Australia

In Australia, average UPF intake was 38.8% of total energy [SE: 0.2] ^(31)^. In unadjusted models, younger age, male gender, second/third/fourth household income quintiles (the combined income of all household members), a lower education level, a lower Socio-Economic Index for Areas (SEIFA) (greater area-level disadvantage), living in inner regional Australia (vs. living in a major city of Australia) and being Australian born or from an English-speaking country were associated with higher intakes of UPF ^(28–31)^. The associations remained unchanged in adjusted models (adjusted for all other sociodemographic variables and diet quality) ^(31)^, except for gender and rurality/urbanisation, which were no longer significant.

In the adjusted model, 19-30-year-olds consumed 8.3% [95%CI: 6.4, 10·3] more UPF as a proportion of total energy than 51-70-year-olds ^(31)^. The middle quintiles (second, third and fourth) of income had the highest intakes of UPF, with the highest income quintile associated with the lowest intake of UPF, 4.6% less than the second quintile (36.5% [SE:0.7] vs. 41.1% [SE: 0.8] ^(31)^. Individuals with the lowest education level (incomplete high school education or lower) consumed 2.3% [95%CI: 0.2, 4.5] more UPF as a proportion of total energy than individuals with the highest levels of education (tertiary qualification) (39.9% [SE: 0.7] vs 37.6% [SE: 0.6]).

#### Barbados

In Barbados during 2012-13, the average intake of UPF was 41% of total energy (838kcal from UPF/day [95% confidence interval (95%CI): 791, 885]). A younger age was associated with a roughly 20% greater UPF intake (25-44 years old: 889kcal/day [95%CI: 836, 942], 45-64 years old: 737kcal/day [95%CI: 693, 781], but gender and level of education were not significantly associated with UPF intake ^(32)^.

#### Belgium

In Belgium, the average UPF intake was 29.9% [95%CI: 29.0, 30.8] in 2014-15 ^(33,34)^. Unadjusted UPF intakes were higher with a younger age, but there was no difference in UPF intakes across education levels or between genders (males: 29.6% [95%CI: 28.0, 31.0] vs. female: 29.2% [95%CI: 28.0, 30.9]) ^(33,34)^. However in 2004, males had a higher UPF intake than females (32.3% [95%CI: 30.9, 34.3] vs. 28.9% [95%CI: 27.1, 30.2]) ^(33)^.

In adjusted models (adjusted for age, region, BMI and breakfast consumption frequency), age and region of Belgium were significantly associated with UPF intake. 3-5- and 51-64-year-olds consumed around 6-8% more UPF as a proportion of total energy than 6-50-year-olds, with 3-5-year-olds consuming 8.6% [SE: 2.1] more UPF as a proportion of total energy than 35-50-year-olds. Individuals living in Brussels region (+6.1% [SE: 1.2] or Walloon region (+8.1% [SE: 0.8] also had significantly higher UPF intakes than individuals living in Flanders region ^(33)^.

#### Brazil

In Brazil during 2008-9, average UPF intake varied across studies, from 20-30% of total energy (20.5% [95%CI: 20.2, 20.8] ^(38)^, 22.1% ^(35)^, 23.8% ^(36)^, 29.6% ^(37)^). A younger age, female gender, white ethnicity (vs. African-descendent or other ethnicity), higher income, higher education level, urban residence and living in the South and South East regions of Brazil were associated with higher intakes of UPF, or were more likely to be in the highest vs. lowest quintile of UPF intake ((≥ 44% vs. ≤ 13% of TEI) ^(35–37,37)^.

In unadjusted associations, females (21.8% [95%CI: 21.3, 22.2]) consumed 2.6% more UPF as a proportion of total energy than males (19.2% [95%CI: 18.7, 19.7]). 20-39-year-olds consumed 6.3% more energy as UPF than adults 60 years or older (21.3% [95%CI: 20.8, 21.9] vs. 15.0% [95%CI: 14.2, 15.8]), and 10-19-year-olds (26.8% [95%CI: 26.1, 27.6]) consumed over 11% more. Individuals in the highest income tercile consumed over 10% more UPF as a proportion of total energy compared with individuals in the lowest income tercile (26.3% [95% CI: 25.7, 26.9] vs. 15.1% [95%CI: 14.6, 15.5]). Similar magnitude differences in UPF intake were seen across the highest (28.5%) vs. lowest (15.7%) terciles of income in another study (p <0.001) ^(35)^, and when grouped by multiples of minimum wage (MW), with individuals earning >3xMW consuming 30.3% of total energy from UPF, compared to those earning <0.5MW, who consumed 16.3% of total energy from UPF ^(36)^. Individuals living in an urban residence also consumed nearly 10% more total energy from UPF than individuals in rural settings (22.1% [95%CI: 21.7, 22.5] vs. 12.7% [95%CI: 12.3, 13.2]). Those in South and Southeast Brazil consumed 25.7% [95%CI: 25.0, 26.4] and 23.6% [95%CI: 23.0, 24.2] of energy as UPF, respectively, around 10% more than in the North (14.8% [95%CI: 14.3, 15.4]) and North East (14.9% [95%CI: 14.5, 15.3]) regions ^(38)^.

In 2017-18, a younger age was associated with a higher UPF intake. 15-19-year-old males (25.1% [95%CI: 23.3, 26.9]) consumed relatively over 100% more UPF than males aged 80+ (12.7% [95%CI: 10.5, 15.0]), and over a 50% relative increase in 15-19-year-old females (26.2% [95%CI: 24.5, 28.0]) compared with older females (80+) (17.9% [95%CI: 13.7, 22.1]) ^(39)^.

#### Canada

In Canada in 2004, a younger age, male gender, lower education level, rural residence and non-immigrant status were associated with greater unadjusted intakes of UPF ^(40–42)^. Sociodemographics remained significantly associated with UPF intake in the adjusted model (adjusted for age, gender, education, income, physical activity, smoking status, immigration status, and residential area), except for rural residence, which became non-significant ^(41)^. Family income per capita was not significantly associated with UPF intake in either unadjusted or adjusted models from 2004 ^(40,41)^. Non-immigrants consumed over 10% more energy from UPF than non-immigrants (47.8% [SE: 0.3] vs. 36.5% [SE: 0.3], p < 0.05) ^(41)^.

In 2015, the average UPF intake in 2015 was around 46-47% (45.7% [95%CI: 45.0, 46.4] ^(42)^, 46.8% (SE: 0.4) ^(44)^). A younger age, male gender, higher income, lower education level, higher level of food insecurity, rural residence, non-immigrant status and Indigenous identity were associated with higher intakes of UPF, or were more likely to be in the highest vs. lowest tercile of UPF intake (72.8% vs. 24.4%) ^(42–44)^.

Adult males (45.4% [95%CI: 43.8, 47.0] consumed more energy from UPFs than adult females (41.6% [95% CI: 40.2, 43.0]), with no significant difference between males and females aged 55 or older ^(42)^. Income was associated with UPF intake in 2015 (p = 0.0143), with the highest income quintiles more likely to be in the highest vs. lowest tercile of UPF intake ^(44)^. One paper in 2015 reported age and gender associations with UPF intake across levels of food insecurity ^(43)^. Males (47.0% [SE: 3.7]) and females (45.8% [SE: 2.4]) aged 19-64 with severe food insecurity consumed around 8-10% more of total energy from UPFs than males (37.5% [SE: 0.66]) and females (37.6% [SE: 0.56]) aged 19-64 with food security (both comparisons p < 0.05, p-trend across levels of food insecurity: males = 0.009, females = 0.003) ^(43)^.

In trend analyses from 2004 to 2015, UPF intake significantly increased in older (55 or older) males (from 42.5% [95%CI: 41.5, 43.6] to 45.3% [95%CI: 43.9, 46.7]) and females (from 41.7% [95%CI: 40.6, 42.8] to 45.2% [95%CI: 44.0, 46.4]), but significantly decreased in children aged 2-12, adolescent males and females aged 13-18, and adult males and females 19-54 ^(42)^.

#### Chile

In Chile in 2010, the average UPF intake was 28.6% [95%CI: 27.7, 29.6] ^(45)^. A younger age, higher family income, urban residence and living in the Metropolitan region were associated with higher intakes of UPF in both unadjusted and adjusted (for all other sociodemographic variables) models ^(45)^. Gender and the level of education of the head of the household were not significantly associated with UPF intake in either unadjusted and adjusted models.

In the adjusted model, 2-19-year-olds consumed over double the quantity of UPF of adults 65 or older (2-19-year-olds: 38.6% [95%CI: 36.7, 40.6] vs. 65 or older: 18.3% [95%CI: 16.8, 19.8]). There was a linear trend, with 20-49-year-olds consuming 26.7% [95%CI: 25.2, 28.2] and 50-64-year-olds consuming 21.8% [95%CI: 19.7, 24.0] of total energy from UPFs (p-trend < 0.001) ^(45)^. There was also a linear trend in UPF intake across family incomes. Individuals from families with the highest incomes consumed over 4% more of total energy from UPFs than individuals from families with the lowest family incomes (≥6xMW: 30.1% [95%CI: 27.7, 29.6], 3-5xMW: 30.0% [95%CI: 27.8, 32.2], 2xMW: 28.7% [95%CI: 27.2, 30.3], 1xMW: 25.8% [95%CI: 24.0, 27.6]). Individuals living in urban residences consumed 29.3% [95%CI: 21.9, 25.5] of total energy from UPFs, compared with 23.7% [95%CI: 28.3, 30.4] in rural residences, nearly a 25% greater relative intake. Individuals living in the Metropolitan region of Chile had the highest UPF intakes of all regions (30.2% [95%CI: 28.6, 31.8]), approximately 2-3% greater than other regions, and significantly greater than the South, where the average adjusted UPF intake was 26.7% [95%CI: 24.8, 28.6] ^(45)^.

#### Colombia

In Colombia, average UPF intake was 15.9% of total energy in 2005, spanning from 0.2% to 41.1% across quintiles of UPF intake ^(46)^. A younger age, female gender, higher socioeconomic status (based on the System for the Selection of Beneficiaries of Social Programs (SISBEN) composite index), urban residence and living in Bogotá were significantly associated with higher intakes of UPF in unadjusted and adjusted (adjusted for all aforementioned sociodemographic variables) models ^(46)^.

In the adjusted model, 2-19-year-olds consumed nearly 8% more UPF as a proportion of total energy than adults aged 50 or over (2-19-year-olds: 19.3% [SE: 0.3], 10-19-year-olds: 19.3% [SE: 0.2], 50 or over: 11.4% [SE: 0.4]). Individuals in the highest socioeconomic level (22.8% [SE: 1.0]) consumed nearly twice the quantity of UPF (over 10% more as a proportion of total energy) of individuals in the lowest socioeconomic level (12.7% [SE: 0.3]). There was a small but significant difference between genders, whereby males consumed 15.5% [SE: 0.2] of total energy from UPF compared to 16.2% [SE: 0.2] in females (P = 0.007). Individuals living in urban residences (17.3% [SE: 0.2]) had 4.6% and 6.1% higher intakes of UPF than people from central (12.6% [SE: 0.5]) or rural (11.2% [SE: 0.6]) residences, respectively (both comparisons, p < 0.001). Regionally, the highest intakes of UPF were in residents of Bogotá (21.6% [SE: 0.5]) followed by the Eastern region of Colombia (18.1% [SE: 0.4]). The lowest intakes were in the Atlantic region of Colombia (12.7% [SE: 0.3]), nearly half the levels reported in Bogotá ^(46)^.

#### France

The average UPF intake in France was 31.1% [95%CI: 30.3, 31.9] in 2006-7 ^(47)^, and 30.6% [SD: 15.8] in 2014-15 ^(48)^. In 2006-7, a younger age, a complete high school or greater education level, occupation (management/intermediate profession, self-employed/farmer, manual worker/employee, homemaker or disabled person or other vs. retired persons) and urban residence were associated with higher intakes of UPF ^(47)^. 18-39-year-olds consumed nearly double the amount of UPF as a proportion of total energy than adults aged 60 or older (39.1% [95%CI: 37.8, 40.5] vs. 21.6% [95%CI: 20.4, 22.8]). Homemakers, disabled persons, and other occupations had the highest UPF intake (35.9% [95%CI: 34.1, 37.7]), followed by management or intermediate professions (32.2% [95%CI: 30.9, 33.4]), manual workers or employees (32.7% [95%CI: 31.3, 34.2]) and self-employed individuals or farmers (28.1% [95%CI: 25.1, 31.2]). Retired individuals had significantly lower UPF intake than all other occupations, around a third less, at 22.3% of total energy [95%CI: 21.1, 23.5]. Individuals with complete high school education (32.9% [95%CI: 31.8; 34.1]), completing a technical course (32.2% [95%CI: 30.3, 34.0] or University education (31.9% [95%CI: 30.4, 33.4]) had similarly high intakes, whereas individuals with an incomplete high school education had around 6% lower intake as a proportion total energy (26.5% [95%CI: 24.9, 28.1]), a roughly 20% lower relative intake ^(47)^. Urban residents consumed 3% more UPF as a proportion of total energy than rural residents (31.9% [95%CI: 30.9; 32.8] vs. 28.9 [95%CI: 27.4, 30.4]).

During 2014-15, age, education level, occupation, marital status, food insecurity and rurality/urbanisation were associated with higher intakes of UPF ^(48)^. Individuals in the highest vs. lowest tercile of UPF intake (34.1-78.9% vs. 0.1-20.6% of energy from UPF) were more likely to be younger, have middle or secondary school education, be an employee, manual worker, have an intermediate profession or be inactive, have moderate or severe food insecurity, be single or in an unmarried couple, or live in a city with 100,000 or more inhabitants ^(48)^. Individuals in the lowest vs. highest tercile of UPF intake were more likely to be older, have a primary school education, be retired, a farmer, craftsman, shopkeeper or business owner, have a rural residence, be married or widowed or have food security ^(48)^.

Across both 2006-7 and 2014-15, UPF intake did not significantly differ with gender ^(47,48)^, nor with the region of France in 2014-15.

#### Italy

In Italy in 2010-13, the average unadjusted UPF intake among Italian adults (aged 20-97) was 17.3% of total energy [95%CI: 17.1, 17.6], rising to 25.9% [95%CI: 24.8, 27.0] in children and adolescents aged 5-19. A younger age, female gender, occupation other than being retired, marital status, urban residence and region of Italy were associated with higher intakes of UPF in the adjusted model (adjusted for the aforementioned sociodemographic predictors, education level, smoking, physical activity and disease history) ^(49)^. Education level was not significantly associated with UPF intake.

In the adjusted model, differences in adult UPF intake across levels of sociodemographics varied by around 10% as a relative proportion of UPF intake ^(49)^. 20-40-year-olds consumed 3.1% [95%CI: 1.8, 4.4] more of total energy from UPF than adults aged 65 or older. Females consumed 1.28% [95%CI: 0.68, 1.89] more UPF than males. Individuals who were unmarried (+1.26% [95%CI: 0.37, 2.15]), separated or divorced (+1.88% [95%CI: 0.38, 3.38]) and widowed (+1.16% [95%CI: 0.07, 2.24]) consumed more energy from UPF than individuals who were married. Individuals in North Italy consumed 0.73% [95%CI: 0.14, 1.32] more UPF as a proportion of total energy than individuals in South Italy, but intakes in North or South Italy did not significantly differ to those living in Central Italy. Urban residents consumed 1.64% [95%CI: 0.87, 2.42] more UPF as a proportion of total energy than rural residents ^(49)^, and retired persons consumed significantly less UPF than all other occupations (manual, non-manual, housewife, student or unemployed), nearly 2% less than manual occupations (-1.87% [95%CI: -0.91, -2.83]).

#### Korea

In Korea across 2010 to 2018, the average UPF intake was 24.9% [SE: 0.1] ^(50)^. A younger age, male gender, lower income, mid/high education level and urban residence were associated with higher unadjusted intakes of UPF. All sociodemographic predictors remained significant in the adjusted model (adjusted for all aforementioned sociodemographics), except for household income ^(50)^.

In the adjusted model, there was a linear trend of decreasing UPF with increasing age (p-trend < 0.05). Adolescents (13-19-year-olds) consumed the highest amount of UPF, over double the amount of UPF of adults 65 or older as a proportion of total energy (33.8% [95%CI: 32.9, 34.6] vs. 16.3% [95%CI: 15.8, 16.7]). Individuals with a high school (26.4% [95%CI: 25.9, 26.9]), or college or higher education level (26.3% [95%CI: 25.8, 26.9]) consumed about 10% relative greater UPF intake than individuals with a middle school or lower education level (23.4% [95%CI: 23.0, 23.8]). Males and urban residents consumed 3% more UPF relatively than females and rural residents, respectively (both comparisons: 25.8% [95%CI: 25.5, 26.1] vs. 25.0% [95%CI: 24.4, 25.6]) ^(50)^.

In 2016-18, a younger age (greatest in adolescents), male gender, mid/high education level, living alone and urban residence were associated with higher unadjusted intakes of UPF, or were more likely to be in the highest (43.6%) vs. lowest (6.9%) tercile of UPF intake ^(50–52,81)^. Unadjusted UPF intakes did not significantly vary across household income levels in 2016-18 in two smaller KNHANES samples (n = 7,364, aged 19-64 ^(51)^, and n = 9,188, aged 30-79 ^(52)^). However, UPF intake did significantly vary in a larger sample from 2016-18 (n = 19,216, aged 1 or older) ^(50)^, whereby individuals in the second and third quartiles (26.7% [95%CI: 26.1, 27.2]) or highest quartile (27.2% [95%CI: 26.5, 27.8]) of household income had significantly higher UPF intakes than individuals in the lowest household income quartile (22.0% [95%CI: 20.9, 23.1]). In the only study reporting adjusted (adjusted for aforementioned sociodemographics, smoking, alcohol and physical activity) UPF intakes from 2016-18, where the average UPF intake was 26.8% [SE: 0.3], age, gender and education level remained significantly associated with UPF intake, but household income, household status, marital status and rurality/urbanisation were not significantly associated with UPF intake ^(51)^. Adjusted intakes of UPF in males were 1.4% higher than females (27.6% [SE: 0.4] vs. 26.2% [SE: 0.4]), p = 0.0165). 19-29-year-olds consumed two-thirds more UPF than 50-64-year-olds (34.6% [SE: 0.8] vs. 20.6% [SE: 0.4]), p <0.0001), with a linear trend of decreasing UPF intake with older age (p-trend < 0.0001). Similar to the associations in 2010-18, individuals with a high school education (27.6% [SE: 0.4], p < 0.01), or college or higher education (26.8% (SE: 0.4), p < 0.05), consumed about 10% more UPF relative to individuals with a middle school or lower education level (25.0% [SE: 0.4], p < 0.05) ^(51)^.

Average UPF intake increased over time in Korea, from 23.1% [95%CI: 22.7, 23.5] in 2010-12, to 25.5% [95%CI: 25.1, 25.9] in 2013-15, to 26.1% [95%CI: 25.7, 26.5] in 2016-18 (p < 0.0001). UPF intake significantly increased across all sociodemographic strata (age, gender, rural/urban residence, education level and household income). In particular, 20-49-year-olds increased their UPF intake to the greatest extent, by 5% from 2010 to 2018, from 24.8% [95%CI: 24.3, 25.4] to 29.8% [95%CI: 29.2, 30.4] ^(50)^.

#### Mexico

In Mexico in 2012, the average UPF intake (aged 1 or older) was 29.8% [SE: 0.4] of total energy ^(54)^. A younger age, higher head of household education level, higher socioeconomic status, urban residence and living in the Northern region of Mexico were more likely to be in the highest (64.2% [range: 51.8-100%]) vs. lowest (4.5% [range: 0-11.8%]) quintile of UPF intake (all sociodemographic variable distributions across quintiles p < 0.001) ^(53)^. Sociodemographic variables remained significantly associated with UPF intake after adjustment for all other sociodemographic variables ^(54)^. However, gender was not significantly associated with UPF intake in unadjusted or adjusted models ^(53,54)^.

In one study of adults only from ENSANUT, adults younger than 60 consumed 21.4% of total energy from UPF [95%CI: 18.8, 24.0], compared with 14.2% [95%CI: 10.7, 17.6] in adults 60 or older, a 50% relative increase ^(27)^. In the adjusted model across all age groups, pre-school-aged children (+12.5% [95%CI: 10.9, 14.1]), school-aged children (+3.8% [95%CI: 2.2, 5.4], and adolescents (+3% [95%CI: 1.1, 4.9]) all consumed greater amounts of UPF than adults. Individuals in North Mexico and Central Mexico consumed 8.4% [95%CI: 6.6, 10.1] and 2.7% [95%CI: 1.2, 4.1] more UPF as a proportion of total energy than individuals in South Mexico. Individuals from households with the highest head of household education level (college education) consumed 7.8% [95%CI: 4.3, 11.4] more UPF as a proportion of total energy than individuals from a household with a head of household without any education. Individuals from a household with a head of household with an intermediate education level consumed 1.9% [95%CI: -0.5, 4.3] (elementary), 3.4% [95%CI: 0.8, 6.1] (middle school) and 4.3% [95%CI: 1.1, 7.4] (high school) more UPF as a proportion of total energy than individuals from a household with a head of the household without any education. Individuals in the middle and highest terciles of socioeconomic status (index based on household characteristics and basic goods and services) consumed 4.5% [middle tercile: 95%CI: 2.8, 6.2, highest tercile: 95%CI: 2.5, 6.5] more UPF as a proportion of total energy than individuals in the lowest tercile. Urban residents also consumed 5.6% [95%CI: 4.2, 7.0] more UPF as a proportion of total energy, compared to rural residents ^(54)^.

#### Netherlands

In the Netherlands in 2012-2016, the average UPF intake was 893g of UPF per 2000kcal [95%CI: 879, 907], or 61% of total energy intake ^(55)^. A younger age, middle education level (vs low or high) and urban residence were associated with higher UPF intake, whereas gender was not significantly associated with UPF intake ^(55)^.

Children and adolescents consumed around double the amount of UPF as older adults, and around 30-50% more than younger and middle-aged adults. For example, 4-8-year-olds consumed 1,252g of UPF per 2000kcal [95%CI: 1,217, 1,288], compared with 962g of UPF per 2000kcal [95%CI: 921, 1,003] in 19-30-year-olds, and 632g of UPF per 2000kcal [95%CI: 607, 656] in 71-79-year-olds. Individuals with a middle education level (intermediate vocational or higher secondary education) consumed around 8-10% more UPF relatively than individuals with lower (primary, lower vocational or advanced elementary) or higher (higher vocational or university education) education levels, consuming 939g of UPF per 2000kcal [95%CI: 916, 962], compared with 871g [95%CI: 838, 903] in individuals with a low education level, and 850g [95%CI: 830, 871] in individuals with a high education level. Individuals living in regions with a high degree of urbanisation (≥1,500 addresses/km^2^) consumed 916g of UPF per 2000kcal [95%CI: 891, 942], compared with 876g of UPF per 2000kcal [95%CI: 856, 896] in individuals living in regions with a low degree of urbanisation (<1,000 addresses/km^2^) and 898g of UPF per 2000kcal [95%CI: 868, 928] in individuals living in regions with a moderate degree of urbanisation (1000–1500 addresses/km^2^) ^(55)^.

#### Portugal

In Portugal in 2015-16, average UPF intake was 23.8% of total energy, or 257g [interquartile range: 141, 426] per day ^(57)^. Younger adults consumed more UPF than elderly adults ^(56)^. Other associations were stratified by male and female gender ^(57)^. In unadjusted models stratified by gender, crude UPF intake was significantly higher with a younger age (highest in adolescents), mid-high education level, in single, divorced or widowed individuals, individuals living in a larger household and living in Lisbon and Azores regions of Portugal ^(57)^. All variables remained significantly associated with UPF intake in adjusted (adjusted for age, education level and non-UPF intake) models, except for household status in both males and females, and marital status in females, which became non-significant. Rurality/urbanisation and food insecurity were not significantly associated with UPF intake in unadjusted or adjusted models^(57)^.

In the adjusted models, adolescents (aged 10-17) had the highest UPF intake, with female adolescents consuming 192g [95%CI: 135, 249] more than female older adults, and male adolescents consuming 327g [95%CI: 277, 377] more than male older adults (aged 45-64). Female and male older adults (aged 45-64) consumed 63g more [95%CI: 34, 91] and 51g more [95%CI: 9, 93] than elderly females and males (aged 65-84). The difference in UPF intake across ages was greater in males than females ^(57)^. Females in Alentejo (+50g [95%CI: 9, 90]) and Algarve regions (+36g [95%CI: 1, 70]) consumed more UPF than females in North Portugal, and males from Lisbon consumed 76g [95%CI: 19, 133] more UPF than males in North Portugal. Males and females with the highest level of education (more than 12 years) consumed 68g [95%CI: 12, 124] and 51g [95%CI: 16, 86] more UPF per day than males and females with the lowest level of education (6 years or less), respectively, a roughly 20-25% relative increase. However, a lower education level was associated with a higher UPF intake in children. Single, divorced or widowed males consumed 48g [95%CI: 1, 96] more UPF per day than married males, or males in a couple.

#### Spain

In Spain, the average intake of UPF significantly increased from 1991 to 2008, accounting for 24.4% [SD: 14.0] of total energy in 1991, 25.6% [SD: 16.3] in 1996, 27.5% [SD: 19.2] in 2004, and 31.1% [SD: 19.0] in 2008 ^(58)^.

In 1991, a younger age was inversely related with higher intakes of UPF (ρ = -0.53, p < 0.0001) ^(59)^. In 2008-10, Individuals in the highest (42.8% [SE: 0.2]) vs. lowest (8.7% [SE: 0.1]) quartile of UPF intake were more likely to be younger and have a primary level education, compared with individuals in the lowest vs. highest quartile of UPF intake, who were more likely to have no formal education and be living alone ^(60)^. Gender proportions did not significantly differ across quartiles of UPF intake.

In the adjusted model (adjusted for year of cohort, age, gender, BMI and total energy intake) across 1991 to 2008, a younger age (-0.15% [SE: 0.01] per year of age) and female gender (1.1% [SE: 0.3] greater UPF intake than males) had significantly higher UPF intakes ^(58)^. UPF intake also varied across regions. In 2008, UPF intake was more than 30% of total energy in all regions, but 5% higher in North Spain (36.0% [SD: 18.3]) than South or Central South Spain (South: 31.3% [SD: 18.3], Central South: 30.2% [SD: 17.1]) ^(58)^.

#### Switzerland

In Switzerland during 2014-15, the average UPF intake was 28.7% of total energy [IQR: 19.9, 38.9] ^(61)^. In unadjusted associations, a younger age, living in the German-speaking region (vs. French- or Italian-speaking regions) and Swiss nationality (vs. non-Swiss) were significantly associated with a higher intake of UPF ^(61)^. After adjustment for other sociodemographics including income, male gender also became significantly associated with higher UPF intake. Household size and education level were not significant in adjusted or unadjusted models.

Across ages, unadjusted median UPF intakes were 8.5% higher as a proportion of total energy in 18-29-year-olds (34.8% [IQR: 24.5, 45.0], compared with 65-75-year-olds (26.3% [IQR: 17.1, 35.0], p = 0.001). Individuals from German-speaking region (29.6% [IQR: 20.9, 39.6]) also consumed 1.5-2.5% more UPF than other regions (French-speaking: 27.2% [IQR: 17.7, 37.1]; Italian-speaking: 28.0% [IQR: 16.9, 39.4], p = 0.002). Swiss nationals consumed relatively around 12% more UPF as a proportion of total energy than non-Swiss nationals (29.2% [95%CI: 20.3, 39.0] vs. 26.1% [IQR: 17.5, 37.1], p = 0.002) ^(61)^.

#### UK

In the UK, average UPF intake was 51.3% [SD: 13.1] in 2008-9 (≥19 years) ^(62)^, 53.1% across 2008-12 (≥18 years)^(64)^, 56.8% [SE: 0.2] across 2008-14 (≥1.5 years)^(65)^, 54.3% [SE: 0.4] across 2008-16 (19-96 years) ^(66)^, and 54.0% in 2014-16 (≥4 years) ^(67)^. From 2008 to 2016, UPF intakes have been relatively consistent, with no significant linear trends in UPF intake across sociodemographic strata ^(63)^.

In 2008-9, a younger age was significantly associated with higher intakes of UPF (−0.16% [95%CI: −0.24 to −0.09] per year of age), but gender, occupational social class (routine and manual or intermediate vs. managerial and professional) and household status (living with other adults or living with children) were not significantly with higher intakes of UPF in adjusted models (adjusted for the aforementioned sociodemographic variables and food preparation skill/behaviours) ^(62)^. Across 2008-12, a younger age (−0.18% [95%CI: −0.21, −0.14] per year of age) and male gender (1.38% [95%CI: 0.09, 2.67]), but not occupational social class, were significantly associated with higher intakes of UPF in adjusted models (adjusted for aforementioned variables and percentage of energy intake from alcohol) ^(64)^. Across 2008-14, younger ages were significantly associated with higher intakes of UPF, with the highest intakes in 11-18-year-olds (68.0% [SE: 0.4]) then 1.5-10-year olds (63.5 [SE: 0.34]) and lowest in adults (19-64: 54.9% [SE: 0.4]) and the elderly (≥65: 53.0% [SE: 0.52]) (all p < 0.001 with 1.5-10 years as reference) ^(65)^. In 2014-16, children (65.7% [95%CI: 64.2, 67.1]) and adolescents 67.1% [95%CI: 65.7, 68.5]) consumed greater quantities of UPF than adults (54.0% [95%CI: 53.0, 55.0]) ^(67)^. Across 2008-16, a younger age, male gender, white ethnicity (vs. non-white ethnicity), lower occupational social class and living in Northern Ireland were associated with higher intakes of UPF as a proportion of TEI ^(66)^. 19-29-year-olds (59.2% [SE: 1.3]) consumed around 8% more UPF as a proportion of total energy than adults aged 60 or older (51.5% [SE: 0.5]), and 5% more than 30-59-year-olds (54% [SE: 0.4]). Males consumed 3% more UPF as a proportion of energy intake than females (55.9% [SE: 0.6] vs. 52.8% [SE: 0.4]). White ethnicity was associated with a 10% higher intake of UPF as a proportion of total energy than other ethnicities (55.4% [SE: 0.4] vs. 45.4% [SE: 1.2]). Individuals in routine and manual occupations consumed 57.3% of energy from UPF, compared with 53.4% [SE: 0.8] in intermediate occupations, 53.8% [SE: 0.7] in lower managerial and professional occupations, and 50.3% [SE: 0.7] in higher managerial and professional occupations (p-trend < 0.05). Individuals living in Northern Ireland consumed 58.7% of total energy [SE: 0.8] from UPF, compared with 51.7% [SE: 0.6] in individuals living in the South of England (including London), who had the lowest intakes of UPF. Average UPF consumption in North England, Central England/Midlands, Scotland and Wales was around 55-57% of total energy ^(66)^.

#### US

In the US, average UPF intake increased from 53.5% [95%CI: 52.5, 54.6] in 2001-2 to 57% [95%CI: 55.0, 58.9] in 2017-18 ^(69)^.

In 1988, individuals in the highest (5.2 to <29.8 times/day) vs. lowest quartile (0 to <2.6 times/day) of frequency of UPF intake were more likely to be younger, male, non-Hispanic white, and were less likely to be Mexican or other ethnicity, have an education below high school level or have a high income/poverty ratio (≥350% the poverty level) ^(68)^.

Across 2005 to 2018, younger age, non-Hispanic white or black ethnicity, a lower income/poverty ratio (<350% of the poverty level) were significantly associated with a higher UPF intake, or were more likely to be in the highest quantile of UPF intake ^(70–73,75–79)^. Income was not significant in one study from 2013-14 ^(79)^. Hispanics and other ethnicities including non-Hispanic Asians and non-Hispanic Asian Americans had low UPF intakes, or were less likely to be in the highest quantile of UPF intake ^(70–73,75,76,78,79)^. Non-Hispanic Asians consumed nearly 20% less unadjusted UPF (39.3% [95%CI: 38.1, 40.5]) than non-Hispanic white (57.7% [95%CI: 56.9, 58.5]) or black (60.1% [95%CI: 58.8, 61.3]) ethnicities ^(77)^. Education was also significantly associated with UPF intake, typically with higher UPF intakes with a mid-low education level ^(69–71,77)^, or mid-level education ^(73,75,76)^. One paper stratifying by ethnicity reported higher UPF intakes were seen in non-Hispanic Asian Americans with higher education levels (lowest vs. highest: 32.1% [95%CI: 29.2, 35.1] vs. 39.7% [95%CI: 38.3, 41.1]), whereas higher UPF intakes were seen in non-Hispanic white or non-Hispanic other ethnicities at lower education levels (non-Hispanic white lowest vs. highest: 61.7% 95%CI: 59.9, 63.5] vs. 53.3% [95%CI: 52.2, 54.5]; non-Hispanic other lowest vs. highest: 62.0% [95%CI: 55.9, 68.0] vs. 50.6% [95%CI: 46.7, 54.6]), and higher UPF intakes in non-Hispanic blacks at mid-low education levels ^(77)^. Similarly a higher income/poverty ratio, was associated with a higher UPF intake in non-Hispanic Asian Americans (lowest vs highest: 35.0% [95%CI: 31.9, 38.1] vs. 40.8% [95%CI: 39.2, 42.4]), but associated with a lower UPF intake in non-Hispanic whites (lowest vs highest: 61.1% [95%CI: 59.7, 62.4] vs. 55.8% [95%CI: 54.8, 56.8]) ^(77)^.

Across 2011-16, US-born individuals consumed over 12% more UPF as a proportion of total energy (adjusted for age, gender, family income/poverty ratio, education level and race/ethnicity) than foreign-born individuals (US-born: 57.9% [95%CI: 57.3, 58.5] vs. foreign-born: 45.4% [95%CI: 44.0, 46.8]) ^(76)^. This difference was seen across all sociodemographics (age, gender, income/poverty ratio, education level, ethnicity). Differences between US- and foreign-born UPF intakes were smaller at the highest income/poverty ratio and education levels (around a 10% difference compared with around a 15% difference at low and middle income/education levels), where UPF intake in foreign-born individuals tended to be higher at a higher income/poverty ratio or education level compared to lower levels, whereas UPF intake in US-born individuals tended to be lower at a higher income/poverty ratio or education level compared to lower levels (p-interaction = 0.001). US-born non-Hispanic blacks also consumed 50% more relative UPF as a proportion of total energy than foreign-born non-Hispanic blacks (60.7% [95%CI: 59.7, 61.8] vs. 40.4% [95%CI: 37.0, 43.8], p < 0.001) ^(76)^.

With marital status, unmarried, single or widowed individuals were more likely to be in the highest quartile of UPF intake, compared with being married or living with a partner ^(70,75)^. Across ethnicities, the association was present within non-Hispanic Asian American, non-Hispanic black and Hispanic ethnicities, but not in non-Hispanic white or other ethnicities ^(77)^.

Gender was not significantly associated with UPF intake in most studies ^(71–74,76–79)^; but across 2005-14, females were more likely to be in the highest quantile of UPF intake ^(70,75)^, and across 2011-16, gender proportions across quartiles of UPF intake significantly differed, with no clear relationship ^(75)^.

Adjusted mean intakes (adjusted for age, gender, education, family income/poverty ratio and race/ethnicity) from 2007-2012 show that younger ages (2-9- and 10-19-year-olds) consumed two-thirds of total energy from UPF (2-9: 65.9% [95%CI: 65.0, 66.8], 10-19: 66.8% [95%CI: 65.9, 67.7]), 13-14% more than adults aged 60 or older (52.8% [95%CI: 51.9, 53.7]). 20-39-year-olds also consumed nearly 60% of total energy from UPFs (59.5% [95%CI: 58.7, 60.3]), and 40-59-year-olds over 55% (55.2% [95%CI: 54.1, 56.4]) ^(71)^. Non-Hispanic white (60.2% [95%CI: 59.4, 60.9]) and black ethnicities (60.6% [95%CI: 59.7, 61.5] had the highest adjusted UPF intakes, 5% more than Mexican-Americans (54.8% [95%CI: 53.2, 56.3]) and 8% more than other Hispanics (52.0% [95%CI: 50.3, 53.7]), with all other races having the lowest UPF intake (49.6% [95%CI: 47.3, 51.8]), more than 10% lower than non-Hispanic white or black ethnicities. Individuals with a college level education or higher (55.9% [95%CI: 54.6, 57.2]) consumed nearly 4% less UPF as a proportion of total energy than individuals with a high school education (59.7% [95%CI: 59.1, 60.3]) or individuals with less than a high school education (59.5% [95%CI: 58.4, 60.6]) ^(71)^. Across the lowest to highest levels of family income/poverty ratios, there was a 2% difference in UPF intake as a proportion of total energy (≤1.30: 59.6% [95%CI: 58.6, 60.7] vs. >3.5: 57.7% [95%CI: 56.9, 58.6]).

From 2001 to 2018, adjusted trends in UPF intake (adjusted for age, gender, race/ethnicity, education level and income/poverty ratio) showed that males (+4.3%) and females (+2.7%) increased their UPF intake over time, to 57.2% [95%CI: 55.2, 59.1] in males and 56.8 % [95%CI: 54.6, 59.1] females in 2017-18 (p-trend = 0.001 and 0.002, respectively) ^(69)^. UPF intake increased across all ages (aged 19 or older). Older adults (60 or older) had the lowest UPF intake in 2001-2 (51.7% [95%CI: 49.4, 54.0), but the highest in 2017-18 (57.4% [95%CI: 54.3, 60.4]). UPF intake increased in non-Hispanic black or white individuals (p-trend = 0.001), but not Hispanics (p-trend = 0.081). Hispanics consistently consumed around 5% less UPF than non-Hispanic black or white individuals. Adults of all income levels increased their UPF intake from 2001 to 2018 (p-trends all < 0.05), and UPF intake increased across all education levels (p-trends all < 0.05), with the lowest intake in college graduates across time, about 5% lower than adults with lower education levels ^(69)^.

#### Multinational

Across 22 European countries (Austria, Belgium, Croatia, Cyprus, Czech Republic, Denmark, Estonia, Finland, France, Germany, Greece, Hungary, Ireland, Italy, Latvia, Portugal, Romania, Slovenia, Spain, Sweden, The Netherlands and the UK), gender was not significantly associated with UPF intake when expressed as a proportion of total energy, except for in Portugal (p < 0.01), where females had higher intakes than males (24.5% vs. 19.8%). UPF intakes typically varied by 1-4% between genders within each country.

### Associations by sociodemographic predictor

Sociodemographic associations with UPF intake by country are presented in Table 3.

#### Age

Age was assessed across 17 countries. There was a consistent association of a younger age (in adults, or in adults and children) having higher UPF intakes in all countries, with some studies showing the highest intakes in adolescents. Differences in absolute UPF intake across ages were large, typically between 5-20% as a proportion of total energy, reflecting 15-100% relative differences in UPF intake. Two studies in the US ^(69)^, and Belgium ^(33)^, also reported relatively high UPF intakes in the elderly or in older adults.

#### Gender

Gender was assessed across 32 countries. Most of the national differences in UPF intake between genders were not significant, or varied in significance across studies within the same country (eight countries). Where significant differences were seen in Australia, Korea, Canada, Switzerland and the UK, males consumed around 1-4% more as a proportion of total energy. In Brazil, Colombia, Italy, Portugal and Spain, females consumed around 1-3% more UPF as a proportion of total energy.

#### Race/ethnicity

Three countries assessed race/ethnicity (Brazil, the UK and the US). Significant and large differences in UPF intake were seen across race/ethnicities, with 10-20% absolute differences in UPF intake as a proportion of total energy in UK and US, corresponding to 20-50% relative differences in UPF intake. In Brazil, the lowest quintile (≤ 13% of total energy) of UPF intake constituted 34% white and 64% African-descendent, compared with 57% white and 41% African-descendent in the highest quintile (≥ 44% of total energy) ^(82)^.

#### Income

Income was assessed in six countries (Australia, Brazil, Canada, Chile, Korea and the US). Five countries reported significant associations between income and UPF intake.

A higher income was associated with a higher UPF intake in Chile and Brazil, with 10-15% absolute differences in UPF consumption as a proportion of total energy across the highest and lowest income levels in Brazil, and over 4% absolute differences in Chile, reflecting a 15-100% relative increase in UPF intake with higher income.

In Australia, the second, third and fourth income quintiles had 2-3% higher adjusted absolute intakes of UPF as a proportion of total energy, compared with the lowest quintile ^(31)^. In the US, there was a 2% difference in adjusted UPF absolute intake as a proportion of total energy across income:poverty levels, increasing with lower income:poverty levels ^(71)^. The association between income and UPF intake in the US differed based on ethnicity. In Korea, there was a 4-5% crude difference in UPF intake between low and mid-to-high incomes across 2010-18, but adjusted mean intakes were non-significantly different ^(50)^. In Canada, income levels significantly differed across terciles of UPF intake in 2015, but no difference was seen in 2004.

#### Education level

Education level was assessed in 15 countries, with no assessment in individual studies from Colombia or the UK. There was a significant association between education level and UPF intake in 10 countries, with no significant association in Barbados, Belgium, Chile, Italy or Switzerland.

A lower education level was associated with a higher UPF intake in Australia (adjusted: 2.3% absolute difference, 6% relative difference) and Canada (adjusted: 1.8% absolute difference, 4% relative difference), and in the US after 2001. Some US studies showed higher UPF intakes with mid-low vs. high education levels (adjusted: 4% absolute difference, 7% relative difference) ^(71)^, or middle education levels ^(73,76)^. The association between education level and UPF intake in the US also differed based on ethnicity.

In the Netherlands, a middle education level (intermediate vocational education, higher secondary education) was associated with about an 8-10% greater relative UPF intake than lower or higher education levels (in grams of UPF per 2000kcal). In France, a mid-high education level had the highest UPF intakes in 2006-7, with the lowest intakes in the lowest education level (6% absolute difference, 20% relative difference). By 2014-15, middle education levels were more likely, and low education levels less likely, to be in the highest UPF intake quartile in France. Similarly in Spain, low education levels (no formal education) were less likely to be in the highest quartile of UPF intake, and mid-level (primary) education were more likely be in the highest quartile of UPF intake, with similar proportions of individuals with a high education level (secondary or higher education) across quartiles of UPF intake ^(60)^.

A higher education level was associated with a higher UPF intake in Brazil (across quartiles), Korea (adjusted: 2-3% absolute difference, 10% relative difference), Mexico (adjusted: 7.8% absolute difference, 25% relative difference) and Portugal (adjusted: 20-25% relative difference in grams), ranging from 2-8% higher as an adjusted proportion of total energy from UPF.

#### Socioeconomic status

Socioeconomic status, assessed via indices of occupation, social class or deprivation was assessed in six countries, with significant, but varying associations with UPF intake in all six countries.

A higher socioeconomic status was associated with higher UPF intake in Colombia (adjusted: 10% absolute, 80% relative) and Mexico (adjusted: 4.5% absolute, 15% relative).

A lower compared with higher socioeconomic status was associated with a higher UPF intake in Australia (adjusted: 2.5% absolute difference) and in the UK across 2008-2016 (based on occupational social class; 7% absolute difference, 14% relative difference) ^(66)^, but not in 2008-9 ^(62)^, or across 2008-12 ^(64)^.

Based on occupation in Italy and France, retired individuals had the lowest UPF intakes (Italy adjusted: 1.9% lower absolute intake than manual occupations, 10% lower relative intake; France in 2006: 6-13% lower absolute intake, 20-40% lower relative intake), with broadly similar intakes or higher proportions in the highest terciles of UPF intake in manual occupations, non-manual occupations, unemployed individuals or students (proportions of professional executives were similar across terciles of UPF intake in France in 2014-15).

#### Food insecurity

Food insecurity was assessed in three countries (Canada, France and Portugal). Higher levels of food insecurity were associated with a higher UPF intake in Canada (10% absolute difference, 20-25% relative increase in UPF across age-gender groups) and more likely to be in the highest tercile of UPF intake in France. Food insecurity was not significantly associated with UPF intake in Portugal.

#### Marital status

Marital status was assessed in five countries, with significant differences reported in four countries (France, Italy, the US and in Portuguese males), and tended to find unmarried, single, separated or divorced individuals had higher UPF intakes, or were more likely to be in the highest quantiles of UPF intake, compared with married individuals or individuals living together. Marital status was not significant in Korea, after adjustment for other sociodemographic factors, or in Portuguese females.

#### Household status

The number of individuals in the household was assessed in five countries, with a significant association with UPF intake in one country. In Spain, people living alone were less likely to be in the highest quartile of UPF intake, but household status was not significant in Switzerland, the UK, Korea or Portugal after adjustment for other sociodemographic factors or health behaviours.

#### Rural/urbanisation

The level of urbanisation was assessed in 11 countries, with higher UPF intakes typically being reported in more urban than rural residences in eight countries: Brazil (10% absolute difference, 80% relative difference), Colombia (6% absolute difference, 50% relative difference), Chile (6% absolute difference, 25% relative difference), France (3% absolute difference, 10% relative difference), Italy (1.6% absolute difference, 10% relative difference), Korea in 2010-2018 (0.8% absolute difference, 3% relative difference), Mexico (5.6% absolute difference, about 20% relative difference) and the Netherlands (5% relative difference). Urbanisation was not significant in Korea during 2016-2018, after adjustment for other sociodemographic factors and health behaviours.

Individuals from rural residences were more likely to be in the highest tercile of UPF intake in Canada in 2015, but there was no significant association in 2004 after adjustment for other sociodemographic factors and health behaviours. Living in inner regional Australia was crudely associated with a higher UPF intake, and living in a major city associated with a lower intake, but did not remain significant after adjustment for other sociodemographic factors. Urbanisation was also not significantly associated with UPF intake in Portugal.

#### Region of country

Region of country was assessed in 11 countries, with ten countries demonstrating regional differences in UPF intake, typically varying by 5-10% as a proportion of total energy, or a 25-75% relative difference in UPF intake: Belgium (adjusted: 6-8% absolute difference, 20-25% relative difference), Brazil (10% absolute difference, 75% relative difference), Chile (adjusted: 4% absolute difference, 13% relative difference), Colombia (adjusted: 9% absolute difference, 70% relative difference), Italy (adjusted: 0.1% absolute difference, 9% relative difference), Mexico (adjusted: 8% absolute difference, 33% relative difference), Portugal (20% relative difference in grams), Spain (in 2008: 5% absolute difference, 20% relative difference), Switzerland (2.4% absolute difference, 8% relative difference), UK (7% absolute difference, 14% relative difference). There was no significant difference in UPF intake across regions of France.

#### Immigrant status / Country of birth

Immigrant status or country of birth was assessed in four countries. Home-born vs. foreign-born individuals in the US (adjusted: 12% absolute difference, 28% relative difference), home-born and English-speaking country born individuals vs. other individuals in Australia (6-9% absolute difference, 20-30% relative difference), Swiss nationals vs. non-Swiss nationals in Switzerland (3% absolute difference, 12% relative difference) and non-immigrants vs. immigrants in Canada (11% absolute difference, 30% relative difference), had around 3-13% higher absolute intakes of UPF as a proportion of total energy, or a 10-30% higher relative UPF intake.

#### Indigenous identity

Indigenous identity was assessed in Canada only. Individuals with Indigenous identity were more likely to be in the highest tercile of UPF intake ^(44)^.

### Associations by country-level income and adjusted analyses

When considering upper-middle income countries (Brazil, Colombia and Mexico), a higher socioeconomic score was associated with higher UPF intake in Colombia and Mexico, a higher income with higher UPF intake in Brazil, higher education level with higher UPF intake in Brazil and Colombia, and a higher UPF intake in all three countries in a more urban residence.

When considering multivariate adjusted associations only, 19 studies across 13 countries reported sociodemographic associations adjusted for other sociodemographic characteristics and health behaviours. The majority of the significant crude associations between sociodemographic variables and UPF intake remained significant with adjustment for other variables. Further details are provided in the supplementary materials.

### Assessment of methodological quality

Risk of bias scores for each study are presented in table 1. Most studies scored a 5 or 7 out of 10 (average: 5.7/10), depending on whether adjustment was made for one or more sociodemographic variables. Risk of bias scores were higher (indicating a lower risk of bias) for studies performing adjustment for other sociodemographic factors.

## Discussion

This systematic review included 55 nationally representative studies, spanning 32 countries and three decades of dietary intake. Average UPF intake varied greatly across countries, from 14-16% of total energy intake in Italy, Romania and Colombia, to 61% in the Netherlands. Intakes also varied greatly within countries, with several sociodemographic factors being independently associated with UPF intake.

Age (being highest in either younger adults, or adolescents and children) demonstrated a consistent inverse association with UPF intake, with a large magnitude difference in UPF intake with age. Other sociodemographic characteristics associated with large magnitude differences in UPF intake across strata included race/ethnicity, income, country of birth, region of the country, rural/urban living and food insecurity. Despite only a few studies reporting on race/ethnicity, ethnic differences in UPF intake were large, with 10-20% absolute differences as a proportion of total energy. Similar magnitude differences were seen in the small number of countries reporting UPF intake based on country of birth or food insecurity, except no difference in UPF intake seen in Portugal across levels of food insecurity.

Living in an urban residence and being unmarried, single, separated or divorced were typically associated with a higher UPF intake, whereas education level, income and socioeconomic status showed varying directions of association with UPF intake, depending on country. Gender was generally not significantly associated with UPF intake in most countries, and neither was the number of individuals in the household. At least one multivariate adjusted association was reported in 13 countries, showing largely unchanged estimates from the crude associations. These findings indicate that the significant and large differences in UPF intake across levels of sociodemographic variables are independent of other sociodemographic variables.

The results from this systematic review confirm and contrast with the findings from previous reviews. Similar to the results in this systematic review, a systematic review that included non-nationally representative samples suggested minimal differences in UPF intake with gender, but with higher intakes in younger ages ^(15)^. One review suggested links between a younger age, urban residence, male gender, lower education level, lower household income and food insecurity with higher UPF intake ^(13)^. Another review suggested links between age, gender, education and income with UPF intake, and also a varying association of socioeconomic status depending on country-level income ^(14)^. This review also identified urban living as an important predictor of greater UPF intake, which is also in line with the findings from global UPF sales and household purchases ^(3,5)^. But, male gender was not consistently associated with higher UPF intakes, and the association between education level and UPF intake varied across countries. Indeed, in UMICs, higher education levels, incomes and socioeconomic status tended to have higher UPF intakes, but the association between education level and UPF intake across HICs varied. In the US, differences in UPF intake across levels of education and income also showed contrasting associations across ethnic groups, and in Portugal, the association between education level and UPF intake differed based on age. These findings indicate the need for more detailed assessments in other countries to tease apart the sociodemographic inter-relationships with UPF intake. Race/ethnicity, region of the country and country of birth were all significant predictors of UPF intake in this systematic review, but had largely been unconsidered in previous reviews. Given the lack of studies assessing the association between food insecurity and UPF intake, and the large differences in food insecurity across ethnic groups ^(83)^, these highlight important and understudied sociodemographic associations for the consumption of UPF intake.

Meta-analyses demonstrate significantly increased risks of poor health with each 10% increment in UPF intake as a proportion of total energy in the diet, including a 15% increased risk of all-cause mortality ^(84)^, 7% higher risk of overweight, 6% higher risk of obesity, 5% higher risk of abdominal obesity ^(85)^ and 15% higher risk of type 2 diabetes ^(86)^. Meta analyses comparing the highest vs. lowest quantiles of UPF intake from prospective cohort studies also show a 25% higher risk of all-cause mortality, 29% higher risk of cardiovascular disease incidence and mortality, 11% higher risk of overweight or obesity, 20% higher risk of depression ^(9)^ and 23% higher risk of hypertension ^(87)^. The magnitude of the absolute differences in UPF within several sociodemographic variables reported in this systematic review were typically in the range of 5-20% of total energy intake (e.g. across age, race/ethnicity, income, country of birth and region of the country) and independent of other sociodemographic variables. These independent differences in UPF intake correspond with the differences in UPF exposure that are associated with significantly increased risks of non-communicable disease reported in meta-analyses. The findings from this systematic review highlight existing health inequalities across sociodemographic subgroups associated with increased UPF intake, and future sociodemographic differences in non-communicable disease incidence. Furthermore, by considering each of the independent sociodemographic associations produces striking differences in mean UPF intake across specific sociodemographic populations. For example in Mexico, the average male adult, living in a rural residence in the south of the country, with a low socioeconomic status and from a household with no head of household education consumes 39.3% less UPF as a proportion of total energy than the average female pre-school child, living in an urban residence in North Mexico with a medium-high socioeconomic status and from a household with a head of the household with a college graduate education (25.2% vs. 64.5%) ^(54)^. Given the growing epidemics of obesity and cardiometabolic disease, these results present an alarming picture regarding the incidence of future adiposity-related cardiometabolic disease.

Notably, no studies included in this review were from Africa or Asia, accounting for over half of the global population. Non-nationally representative samples from Asian countries also demonstrate sociodemographic associations with UPF intake, supplementing the global picture of national UPF intake patterns. With almost a fifth of the global population, results from the China Health and Nutrition Survey across 1997-2009 (CHNS; 12,451 adults sampled using a random, complex multistage methodology across nine of 31 Chinese provinces, but not considered to be nationally representative ^(88)^) showed that the highest UPF consumers (≥50g UPF/day) vs. non-consumers vs. (0g/day) were more likely to be male, have a higher income, higher education level and live in an urban residence ^(22)^, similar to the UMICs in this review. Age was not significantly associated with UPF intake. However, another CNHS study reported similar findings, except age was positively associated with UPF intake, in contrast to the findings here, ^(89)^. Other Asian samples report differences in UPF intake with a younger age, higher education level and race/ethnicity, with variable gender associations. In Jakarta, Indonesia, a younger age was associated with increased UPF intake ^(90)^, and in a multicentric study from Iran, being under 40 (vs. 40 or older) and having a higher level of education were associated with higher intakes of UPF, but there was no difference in UPF intake with gender ^(91)^. In middle-aged Japanese adults, people who were never married, living alone, in regular full-time work and with a lower income had higher UPF intakes, but no difference was seen with age, gender or number of children ^(92)^. In the Singaporean multi-ethnic cohort study, the highest vs. lowest quartiles of UPF intake (85.9% vs 51.8% of total energy) were more likely to be younger, male, be of Malay or other ethnicity, and less likely to be of Indian or Chinese ethnicity ^(23)^. In the Taiwan, a higher UPF intake was seen with a younger age and a higher education level, but gender was not significant ^(24)^. Improving the understanding the sociodemographic patterns of individual-level UPF intake in African and Asian nations represents a key research focus for understanding the global implications of UPF consumption.

### Strengths and limitations

This review builds upon previous reviews by systematically reporting sociodemographic predictors of individual-level UPF intake from nationally representative samples, reducing biases resulting from convenience sampling, increasing the generalisability of results to each nation. The use of food consumption surveys strengthens the confidence in assessing individual-level UPF intake, compared with household purchase data. This review reported all sociodemographic predictors from included studies, including those which were not associated with UPF intake, and provided absolute and relative quantitative estimates of differences in UPF intake across sociodemographic strata. Multivariate analyses were conducted in several countries in this review, allowing for the identification of independent sociodemographic associations, separate to other sociodemographic characteristics and health behaviours.

However, some limitations must be considered. First, the lack of complete reporting of all sociodemographic predictors precludes determining the most important sociodemographic determinants of UPF intake. Within each country, most studies considered age and gender, but other sociodemographic predictors were not consistently reported across all studies, and multivariate adjustments in assessing associations were not performed in all countries. In addition, different metrics and stratifications were used across papers to report the same sociodemographic factor (e.g. different methods of assessing socioeconomic status), limiting comparability. Other sociodemographic influences were also not considered, such as work shift pattern. Second, the use of food consumption surveys was a criterion for inclusion, but are time intensive and costly. Combined with the requirement for nationally representative sampling methodology, the inclusion criteria biased towards including higher income countries with the resources to conduct such studies. Meaning, many countries were not included in this review, particularly those from middle- and low-income countries. Third, UPF intake was self-reported, as was the assessment of sociodemographic variables, which may have introduced recall bias or bias from misreporting. The dietary assessment method used to assess UPF intake is an important factor. Accurate classification requires extensive details on a food item, which is typically more than what is provided in an FFQ. FFQs also do not allow for the ability to categorise similar foods into different NOVA groups (for example, a single item in the FFQ for all breads or lasagne). Multiple-day, 24-hour recalls or food diaries have greater potential to identify UPFs and estimate habitual UPF intake from detailed, open-ended, questions, connected to a database of several thousand food items. All but one study in this analysis used 24-hour recalls or food diaries with an extensive food database coded into NOVA to estimate UPF intake, and most assessed intakes over multiple days. Only one cohort assessed UPF intake with a standalone FFQ. This reduced the risk of misclassification that may occur with less detailed FFQs. However, misclassification is still possible, and the classification of specific foods into NOVA processing groups may vary across studies from coding error. Some studies used a single 24-hour recall, which, whilst providing more granular detail than an FFQ, may not accurately reflect habitual intake. Fourth, despite the NOVA classification being used extensively in research and implemented in national dietary guidelines or health organisation recommendations, there is still disagreement regarding the utility and validity of measuring UPF intake using the NOVA classification. Fifth, bias may have been introduced through excluding participants with missing data for complete case analyses in the adjusted models, or in reports of unadjusted cohort characteristics, depending on the specific outcome of interest in the study. However, agreement amongst samples was high, and the consistency within each country indicates that the impact of exclusion bias across samples was minimal. Most studies did not compare the characteristics of excluded and included samples, resulting in lower risk of bias scores, making it unclear as to the extent of this bias. Adjustment in multivariate models for confounding factors can allow for the determination of independent associations, but not all studies considered all potential sociodemographic factors in multivariate models. Furthermore, some studies also adjusted for non-sociodemographic factors, such as health behaviours, diet quality or BMI, which may bias the estimates for sociodemographic variables. Fifth, the methodological rigour required to meet the inclusion criteria of this systematic review increased the certainty of the evidence examining the relationship between sociodemographic factors and individual-level UPF intake during specific times or across time in nationally representative samples. However, it is not possible to ascertain the extent to which differences within surveys that span different time periods are due to bias, changes in measurement methodology, different analytical approaches, or from genuine changes in UPF intake. Finally, the narrative synthesis limits the ability to determine overall associations within countries and across sociodemographic variables. It was not possible to synthesise papers quantitatively, due to differences in the reporting of sociodemographic associations (e.g. reporting mean UPF intakes or associations across quantiles of UPF intake), and whether papers analysed a single cross-sectional sample, repeated cross-sectional samples, or trends over time.

### Implications and further research

The systematic assessment of sociodemographic determinants of UPF intake in this review indicates that UPF intake is unevenly distributed within nations, varying based on previously identified factors such as age and income, but also with previously unconsidered factors such as race/ethnicity or by region of each country. Furthermore, sociodemographic predictors such as gender appear to be less important than previously suggested, and others, such as education or income, are more nuanced than previously suggested. The results here have implications for health policy and research, indicating that certain groups may obtain greater benefit from policy action and targeted/directed interventions. Importantly, the associations between UPF intake and sociodemographic factors are likely to be a reflection of social injustice, and the adverse associations linked with UPF a result of such inequalities. These findings indicate a need to consider social, cultural and geographical influences on UPF intake, and how barriers to reducing UPF intake and accessing MPF may vary across sociodemographic populations within each country. Whether a given food may be considered as UPF can vary across countries, depending on the typical processing method. For example, breads are typically considered as PF in Australia, but as UPF in the UK. Such cultural differences need to be taken into account when considering public health interventions regarding UPF intake. Little is still known about the actual consumption of UPF within many populations ^(93)^. There is a need for more nationally representative samples assessing individual-level dietary intake that perform multivariate adjustments of a wider range of sociodemographic predictors of UPF intake, particularly in middle- and low-income countries, and in Africa and Asia.

## Conclusion

Average UPF intake varies greatly across countries, but within each country, a number of sociodemographic variables are independently associated with UPF intake, including age, race/ethnicity, marital status, education level, income, rural/urbanisation and region of the country. These are likely a reflection of social injustice. Gender and household status were largely not significantly associated with UPF intake. The magnitude of the differences in UPF intake across sociodemographic levels are comparable to the magnitudes associated with increased risks of obesity, cardiometabolic disease and all-cause mortality, highlighting the importance of policy action and interventions to minimise the health inequalities relating to social injustice.

## Supplementary Material

Supplementary Material

## Figures and Tables

**Table 1:** Studies included in the systematic review, and study-level associations of sociodemographic predictors and UPF intake.

Country	Author, year	Cohort	Sampling method	Year(s) of analysis	Sample size	Diet assessment method	Average UPF intake	Sociodemographics associated with higher UPF intake	Sociodemographics not associated with higher UPF intake	Risk of bias (/10)
**Australia**	Grech, 2022	NNPAS within the Australian Health Survey (AHS)	Complex, stratified, multistage probability cluster sampling design based on the selection of strata, households and people within households	2011-2012	9,341, aged >=19	Two 24-hour nonconsecutive dietary recalls by trained interviewers using a modified validated USDA Automated Multiple-Pass Method.	40.4% (%kcal)	Unadjusted: Younger age, male, lower socioeconomic index for area (SEIFA, Index of Relative Socio-economic Disadvantage), lower education level, Australian born, living in inner regional Australia (lower intake in major cities).		5
	Machado, 2020				7,411, aged >=20		38.9% (%kcal) (Range: 0-100)	Unadjusted: Younger age, lower socioeconomic index for area (SEIFA), Australian born or from an English country, living in inner regional Australia (lower intake in major cities). Less likely to be higher educated or live in a major city.	Gender	5
	Machado, 2019				12,153, aged >=2		42% (%kcal)	Unadjusted: Younger age		5
	Marchese, 2021				8.209, aged >=19		38.8% (%kcal) (SE: 0.2)	Unadjusted: Younger age, male, Australian-born, greater area-level disadvantage (SEIFA), lower education level, second/third/fourth household income quintile, living in inner regional Australia. Fully adjusted: Younger age, Australian- or English-speaking-country-born, greater area-level disadvantage (SEIFA) lowest quintile (vs. highest quintile), lower education level, second/third/fourth income quintiles (vs. first (lowest) quintile).	Adjusted: Gender and rural/urbanisation	7
**Barbados**	Harris, 2022	BNSS within HotN study	Multistage probability sampling, recruitment and data collection	2012–13	359, aged 25–64	Two non-consecutive interviewer-led 24-h recall using AMPM adapted	40.5% (%kcal), 838.1kcal/day [95% CI:791.0, 885.3]	Unadjusted: Younger age	Gender, education level	5
**Belgium**	Vandevijvere, 2018	Food Consumption Survey (FCS)	Multistage stratified sampling	2004 and 2014–2015	2004: 3,083, aged >=15; 2014-15: 3,146, aged 3–64	10-64-year-olds: two 24-hour nonconsecutive dietary recalls. 3-9-year-olds: two self-administered non-consecutive one-day diet diaries and interview with parent or legal guardian	2014-15: 29.9% (%kcal) [95%CI: 29.0, 30.8], excluding misreporters (n=818): 32.6% [95%CI: 31.0, 33.4]	2004 Unadjusted: Males. 2014-15 Unadjusted: Younger age. Adjusted: youngest (3-5) and oldest (51-64) age, living in Brussels or Walloon (vs. Flanders region).	2014-15 Unadjusted: gender, education level	6
	Vandevijvere, 2020			2014-2015	3,146, aged 3-64		29.9% (%kcal) [95%CI: 29.0, 30.8]	Unadjusted: Younger age	Gender, education level	5
**Brazil**	Verly-Jr, 2021	Household Budget Survey (HBS)	HBS: Multistage cluster sampling, stratified by geographic location and economic level. Two-stage sampling process. National diet survey: Random selection of 25% of households from HBS.	2008–2009	32,749, aged >=10	Two 24-hour nonconsecutive food records	23.8% (%kcal)	Unadjusted: Higher income		5
	Louzada, 2017				34,003, aged >=10		22.1% (%kcal), 403.9 kcal/day	Unadjusted: Higher income		5
	Louzada, 2015				30,243, aged >=10		29.6% (%kcal)	Unadjusted: Younger age, female, white ethnicity (vs. African-descendent or other), urban living, higher education level, higher income		5
	Canella, 2018				32,900, aged >=10		20.5% (%kcal) [95%CI: 20.2, 20.8]	Unadjusted: Younger age, female, higher income, urban living, South and South East regions of Brazil		5
	Nilson, 2022			2017-18	aged 30-69			Unadjusted: Age (stratified by gender)		5
**Canada**	Moubarac, 2016	Canadian Community Health Survey– Nutrition (CCHS)	Multistage stratified cluster sampling	2004	33,694, aged >=2	Two 24-hour recalls using adapted USDA AMPM (first recall used in analysis)	47.7% (%kcal) (SE: 0.14), 984.3kcal/day (SE: 7.4)	Unadjusted: Younger age, male, lower education level, rural living	Family income (per capita)	5
	Nardocci, 2018				19,363, aged >=18		45.1% (%kcal) (SE: 0.14), 939.65 kcal/day	Unadjusted: younger age, male, lower education level, non-immigrant, rural living. Adjusted: younger age, male, lower education level, non-immigrant,	Unadjusted: family income (per capita). Adjusted: family income (per capita), rural/urbanisation	7
	Polsky, 2020			2004 and 2015	2004: 33,924, 2015: 20,080 (54,004, aged >=2)		2004: 47.8% (%kcal) [95%CI: 47.3, 48.3], 2015: 45.7% [95%CI: 45.0, 46.4]	Unadjusted: 2004: adolescents and children 2015: adolescents and children. 2004 and 2015: adult males (vs adult females). From 2004 to 2015, decrease in age-sex groups: 2-5, 6-12, adolescent females and males 13-18, adult females and males 19-54. Increase in older males and females 55+.		5
	Hutchinson, 2021			2015	15,909, aged 1-64		Not reported	Adjusted: Higher food insecurity across agegender groups. Within each food insecurity status: no significant difference between gender. Food secure: highest UPF in adolescents, Very food insecure: UPF high in all age-gender groups.		7
	Nardocci, 2020				13,608, aged >=19		47% (%kcal) (SE: 0.41)	Unadjusted: Younger age, male, income, lower education level, rural living, nonimmigrant, Indigenous identity		5
**Chile**	Cediel, 2017	National Dietary Survey, Encuesta Nacional de Consumo Alimentario (ENCA)	Probability sampling by clusters, with stratification and multiple lottery stages	2010	4,920, aged >=2	One 24-hour diet recall using USDA AMPM	28.6% (%kcal) [95%CI: 27.7, 29.6], 545.5kcal/day (SE: 12.8)	Unadjusted and adjusted: younger age, urban living, Metropolitan region, higher family income.	Unadjusted and adjusted: gender, head of household education level	7
**Colombia**	Khandpur, 2020	National Survey of the Nutritional Status of Colombia (ENSI) and National Survey of Demography and Health of Colombia (ENDS)	Stratified, multistage sampling	2005	38,643, aged 2-64	24-hour dietary recall	15.9% (%kcal)	Unadjusted and adjusted: younger age, female, higher socioeconomic status, urban living, Bogota region.		7
**France**	Calixto Andrade, 2021	Étude Nationale Nutrition Santé (ENNS)	Random selection of geographic zones, stratified into regions based on level of urbanisation, with randomly selected households	2006-2007	2,642, aged 18-74	Three non-consecutive interviewer-led 24-hour recalls across weekdays and the weekend	31.1% (%kcal) [95%CI: 30.3, 31.9], 663.3 kcal [95%CI: 640.5, 686.1]	Unadjusted: Younger age, urban living, occupation (in a job), education level (retired and incomplete high school with lowest UPF intake)	Gender	5
	Salomé, 2021	Third Individual and National Study on Food Consumption Survey (INCA3)	Three-stage cluster sampling (geographical units, households and individuals)	2014–2015	1,774, aged 18-79		30.6% (%kcal) (SD:15.8)	Unadjusted: Younger age, education level, in a job, city with ≥100 000 habitants (middle school education more likely higher UPF intake, retired, primary school education level and rural living more likely lower UPF intake), higher food insecurity, single/unmarried couple	Gender, region of France	5
**Italy**	Ruggiero, 2021	Italian Nutrition & Health Survey (INHES), based on Italian Cardiovascular Epidemiologic Observatory Health Examination Survey (CEO-HES)	According to geographical distribution, age, gender and socio-economic profile.	2010-13	9,078, aged 5-97; 8,569, aged 20-97	One 24-hour diet recall	17.8% [95% CI:17.5, 18.1] (Adults: 17.3% [95% CI:17.1, 17.6])	Adjusted: female, younger age, North Italy (vs South), urban living, occupation ((retired with lower intake vs. manual and non-manual), not married (unmarried, separated/divorced, widowed)	Adjusted: education level	7
**Korea**	Shim, 2022	Korea National Health and Nutrition Examination Survey (KNHANES)	Multistage, stratified clustered probability sampling	2010-2018: 2010–2012, 2013–2015, and 2016– 2018	57,423, aged >=1, 20,461 in 2010–2012, 17,746 in 2013–2015, 19,216 in 2016–2018	Single interviewer-led 24-hour recall using the multiple-pass method	24.9% (%kcal) (SE:0.1), 531.4kcal/day (SE:3.3)	Unadjusted: younger age, male, urban living, mid/high education level, low income. Adjusted: younger age, male, urban living, mid/high education level. Over time: all subgroups increase UPF intake, with largest increase in 20-49 year olds.	Adjusted: income	7
	Sung, 2022			2016-2018	7,364, aged 19-64		26.8% (%kcal) (SE: 0.3)	Unadjusted: younger age, male, mid/high education level (high school or higher), urban living, single/separated/divorced, singleperson household. Adjusted: younger age, male, mid/high education level (high school or higher)	Unadjusted: household income. Adjusted: household income, rural/urbanisation, marital status, household status	7
	Shim, 2021				21,075, aged 1+		26.2% (%kcal) (SE:0.2), 549.7kcal/day (SE: 5.7)	Unadjusted: Younger age, male		5
	Shim, 2021				9,188, aged 30-79		23.6% (%kcal) (SD: 17.2)	Unadjusted: Younger age, male, urban living, higher education level	Income	5
**Mexico**	Marrón-Ponce, 2019	Mexican National Health Nutrition Survey (ENSANUT)	Probabilisticbased survey with complex, multi-stage, stratified sampling	2012	10,087, aged >=1	One-day interviewerled 24-hour recall using AMPM	30.0% (%kcal)	Unadjusted: Younger age, urban living, North Mexico (less likely South Mexico), higher socioeconomic score, higher head of household education level	Gender	5
	Marrón-Ponce, 2017				10,087, aged >=1		29.8% (%kcal) (SE: 0.4), 578.7kcal/day (SE: 9.8)	Adjusted: Younger age, urban living, North Mexico, higher socioeconomic score, higher head of household education level	Adjusted: Gender	7
	Oviedo-Solís, 2022				226 adults	Two 24-hour dietary recalls and food frequency questionnaire	Diet recall: 19.2% (%kcal) [95%CI: 17.1, 21.3] Food frequency questionnaire: 19.6 [95%CI: 17.7, 21.5]	Unadjusted: Adults <60 (vs. adults >=60)		5
**Netherlands**	Vellinga, 2022	Dutch National Food Consumption Survey (DNFCS)	Stratified sampling, representative for age, gender, region, urbanisation and education	2012–2016	4,313, aged 1-79	Two non-consecutive 24-hour dietary recalls	61% (%kcal), 893g/2000kcal [95%CI: 879, 907]	Unadjusted: Younger age, moderate education level (vs low or high), higher degree of urbanisation	Gender	5
**Portugal**	Miranda, 2020	National Food, Nutrition and Physical Activity Survey (IAN-AF)	Multistage sampling (stratification into seven geographical regions, random selection of Primary Health Care Units and random selection of individuals)	2015–2016	3,852, aged >=18	Two non-consecutive 24-hour dietary recalls (two non-consecutive 24-hour food diaries for children under 10)	22% (%kcal) (SE:0.38)	Unadjusted: Adults (vs. elderly)		5
	Magalhães, 2021				5005, aged 3-84		23.8% (%kcal), 10.6% (%g)	By gender unadjusted: Younger age (highest in adolescents 10-17), single/divorced/widowed, Lisbon Metropolitan area, Azores region, higher education level, 3-4 or >5 household members (vs. 1-2). Adjusted: younger age (highest in adolescents 10-17), Lisbon Metropolitan area (males), Alentejo and Algarve region (females), single/divorced/widowed (males), higher education level.	(By gender) Unadjusted: Rural/urbanisation, food insecurity. Adjusted: rural/urbanisation, marriage status (females), household status, food insecurity	7
**Spain**	Romero Ferreiro, 2021	Diet and Risk of Cardiovascular Disease in Spain (DRECE)	Stratified cluster sampling	DRECE I 1991	4,679, aged 5-59	Food frequency questionnaire designed and validated for Spanish epidemiological studies	24.4% (%kcal) (SD: 13.9), 370.7g/day (SD: 328.6)	Unadjusted: Younger age		5
	Romero Ferreiro, 2022			DRECE I,II,III and IV 1991, 1996, 2004, 2008	4,679 DRECE I, 928 DRECE II, 1,065 DRECE III, 4,835 DRECE IV		1991: 24.4% %(kcal) (SD: 14.0), 1996: 25.6% (SD: 16.3), 2004: 27.5% (SD: 19.2), 2008: 31.1% (SD: 19.0)	Unadjusted: Canary islands (1991, 1996), Northern region (2004, 2008), lowest intake in the East (1991, 1996, 2004). Adjusted: younger age, female		6
	Blanco-Rojo, 2019	Study on Nutrition and Cardiovascular Risk in Spain (ENRICA) 2008-2010		2008-2010	11,898, aged >=18	Validated computerbased dietary history (DH-ENRICA)	24.47% (%kcal) (SE: 0.17), 385g/day	Unadjusted: Younger age, primary education (no formal education less likely to have higher UPF intake), living with other people	Unadjusted: Gender	5
**Switzerland**	Bertoni Maluf, 2022	Swiss National Nutrition Survey (menuCH)	Multi-stage stratified cluster sampling	2014-15	2085, aged 18-75	Two non-consecutive 24-hour recalls	28.7% [IQR: 19.9, 38.9]	Unadjusted: younger age, German-speaking region (vs. Swiss or French speaking region), Swiss nationality (vs. non-Swiss). Adjusted: younger age, male, German-speaking region (vs. Swiss or French speaking region), Swiss nationality (vs. non-Swiss).	Unadjusted: gender. Unadjusted and adjusted: Household size, education level.	7
**UK**	Lam, 2017	National Diet and Nutrition Survey (NDNS)	Multi-stage probability sampling. Households randomly selected from the UK Postcode Address File	2008–09	509, aged >=19	Consecutive four-day food diary (three or four used in analysis)	51.3% (%kcal) (SD: 13.1)	Adjusted: Younger age	Adjusted: Gender, adults or children in household, occupational social class (intermediate vs managerial & professional / routine & manual vs managerial & professional)	7
	Madruga, 2022			2008-2019	15,643, aged >=1.5		2008-09: 55.3% (%kcal) (SE: 0.6) 2013-14: 58.3% (%kcal) (SE: 0.7) 2018-19: 56.6% (SE: 0.7)		Adjusted linear trends and interaction between linear UPF intake trend and sociodemographic characteristic. No linear trend over time in across sociodemographics	7
	Adams, 2015			2008-12 (years 1-4)	2,174, aged >=18		53.1% [95%CI: 52.4, 53.7]	Adjusted: male gender, younger age	Adjusted: occupational social class	7
	Rauber, 2019			2008-2014 (years 1–6)	9,364, aged >=1.5		56.8% (%kcal) (SE: 0.24)	Unadjusted: Younger age		5
	Rauber, 2020			2008-16 (years 1–8)	6,143, aged 19-96		54.3% (%kcal) (SE:0.4)	Unadjusted: Younger age, male, white ethnicity (vs. non-white), Northern Ireland (lowest intake in South England, including London), lower social class occupation		5
	Nascimento, 2021			2014-16	2,449, aged >=4		Adults: 54.0% (%kcal)	Unadjusted: Younger age		5
**US**	Kim, 2019	National Health and Nutrition Examination Survey (NHANES)	Multi-stage, stratified, clustered, probability sampling design (four stages: counties, blocks, households and the number of people within households)	1988-1994	11,898, aged >=20	Before 2003, one single, in-person recall. After 2003, two 24-hour dietary recalls: one in-person 24-hour recall followed by a telephone-based recall 3–10 days later using USDA AMPM. Diet assessment based on the in-person recall.	4 times/day (Range: 0-29.8)	Unadjusted: Younger age, males, non-Hispanic white, income/poverty ratio (less likely high UPF intake with a higher income/poverty ratio), education level (less likely high UPF intake with less than high school education)		5
	Juul, 2021			2001/2-2017/18 (9 cycles)	40,937, aged >19		2001-2: 53.5% (%kcal) [95%CI: 52.5, 54.6], 2017-18: 57.0 [95%CI: 55.0, 58.9]	Adjusted trends: Younger age in 2001/2, older age in 2017/18. Across 2001/2 to 2017/18: Higher intake in non-Hispanic white or black, lower education level (college graduate vs lower levels (<high school, high school, some college)). 2017/18: Non-Hispanic white or black, lower education level. UPF intake increased significantly among all age groups with no difference in trend. Increase over time in all sociodemographic sub-groups except Hispanics.	Income/poverty ratio	7
	Juul, 2018			2005/6-2013/14 (5 cycles)	15,977, aged 20-64		56.1% (%kcal)	Unadjusted: Younger age, female, Non-Hispanic white or black (Hispanic or other ethnicity less likely to have higher UPF intake), lower family income/poverty ratio, high school graduate (less likely high UPF with college graduate education or higher), marital status (less likely high UPF if married)		5
	Baraldi, 2018			2007/8– 2011/12 (3 cycles)	23,847, aged >=2 (MV analysis = 19,540)		58.5% (%kcal) (SE: 0.3); 1205.4 kcal/day (SE: 7.8)	Unadjusted and adjusted: younger age, mid/low education level, lower family income/poverty ratio, non-Hispanic black (and non-Hispanic white in adjusted). Unadjusted and adjusted linear increase over time in males, adolescents (10-19) and with high school education. Increase in UPF intake in all sub-groups when comparing 2007/8 to 2011/12.	Unadjusted: gender. Adjusted: gender	8
	Steele, 2022			2009/10– 2013/14 (3 cycles)	6,385, aged >=20		55.5% (%kcal) [95%CI:54.6, 56.4]	Unadjusted: Younger age, non-Hispanic white or black, lower income/poverty ratio (less than >3.5x), 12 years education (vs. <12 or >12 years education)	Gender	5
	Yang, 2020			2009/10–2015/16 (4 cycles)	12,640, aged 30-74 without cardiovascular disease or stroke		54.5% (%kcal) (Median) [IQR:45.8, 63.1]		Gender	5
	Steele, 2020				9,416, aged >=6		58.3% (%kcal) (SE:0.4)	Unadjusted: Younger age, non-Hispanic white or black, lower income/poverty ratio (less than >3.5x)	Gender	5
	Zheng, 2020			2011/12-2015/16 (2011–2012, 2013–2014, and 2015–2016)	13,637, aged >=20		54.9% (%kcal) [95% CI: 54.0, 55.7], 1,201 kcal/day	Unadjusted: Younger age, gender (unclear association), non-Hispanic black or white (Hispanic, non-Hispanic Asian less likely to have high UPF intake), widowed/divorced/separated/never married, education level (higher UPF intake with high school (mid) education level), lower annual family income		5
	Steele, 2020				14,663, aged >=20		Not reported	Unadjusted: Younger age, lower income/poverty ratio (less than >3.5x), mid education level, non-Hispanic black, non-Hispanic white or other race (including multiracial), Unadjusted and adjusted: US-born (vs foreign born, true across gender, age, income, education level and race/ethnicity).	Gender	7
	Pachipala, 2022			2011/12-2017/18	20,680, aged >=18		Not reported	Unadjusted: Non-Hispanic black or other race, then Non-Hispanic white. Within each ethncity: younger age, not married (Non-Hispanic Asian American, Non-Hispanic Black, Hispanic), lower educaton level (Non-Hispanic White,Non-Hispanic Other), low/mid education level (Non-Hispanic Black), higher educaton level (Non-Hispanic Asian American), higher income/poverty ratio (Non-Hispanic Asian American), lower income/poverty ratio (less than >=3.5x: Non-Hispanic White) Lowest UPF intake in non-Hispanic Asians.	Within ethnicity: gender, marriage status (Non-Hispanic White, Non-Hispanic Other), education level (Hispanic), income/poverty ratio (Hispanic, Non-Hispanic Other, Non-Hispanic Black)	5
	Buckley, 2019			2013/14	2,212, aged >=6		Quartile 1: 35.3%, Quartile 4: 87.2%	Unadjusted: Younger age, non-Hispanic black or other race/ethnicity, lower income/poverty ratio (less than >=3.5x). Lowest UPF intake in Asian Americans	Gender	6
	Kim, 2019				2,242, aged >=6		Quartile 1: 33.7%, Quartile 4: 89.6%	Unadjusted: Younger age, non-Hispanic black or other race/ethnicity.	Gender, income/poverty ratio	5
**Multinational across Europe (22 countries)**	Mertens, 2021	European Food Safety Authority (EFSA) Comprehensive European Food Consumption Database	24-hour recall, food record or food frequency questionnaire	2003-2017 depending on country	Not reported		14-44%	Unadjusted: Gender not significant except for Portugal (p<0.01)	Gender not significant except for Portugal (p<0.01)	5

**Table 2:** Statistical associations between sociodemographic variables and UPF intake reported in each study. Adjusted estimates are reported where provided, or else unadjusted measures are reported.

Country	Author, year	Statistical measures	Results by sociodemographic variable							
**Australia**	Grech, 2022	Unadjusted means, SE and p value	Age: 19–30 years: 43.9% (SE:0.8); 31–50 years 38.0 (0.4); 51–70 years: 34.5 (0.5); 71+ years: 36.5 (0.7) (p-trend < 0.0001)	Gender: Female: 37.5% (SE:0.4); Male: 38.8 (0.5) (p-trend = 0.047)	SEIFA: Lowest (quintile 1): 40.1% (SE:0.8); Middle (quintile 2–3): 38.4 (0.4); Highest (quintile 5): 35.9 (0.7) (p-trend = 0.0013)	Education: No tertiary education: 40.1% (SE:0.8); Vocational education: 38.4 (0.4); University education: 35.9 (0.7) (p-trend = 0.0013)	Country of birth: Australian-born: 40.3% (SE:0.4); Other English-speaking countries:37.6 (0.8); Other: 31.0 (0.7) (p-trend < 0.0001)	Rural/urbanisation: Major cities: 37.3% (SE:0.3); Inner regional: 40.7 (0.7); Other: 39.3 (1.1) (p-trend < 0.0001)		
	Machado, 2020	Unadjusted p-value across quintiles of UPF intake	Age (20–39 years, 40–59 years, ≥60 years) p < 0.001	Gender (Male, Female) p = 0.493	Years of education (≤9 years, 10–12 years, 10–12 years with graduate degree) p < 0.001	SEIFA (Quintile 1,2,3,4 and 5) p < 0.001	Rural/urbanisation (Major cities, Inner regional, Other) p = 0.002	Country of birth (Australia or English country, Other) p < 0.001		
	Machado, 2019	Unadjusted mean, 95%CI and p value	Young children (2–5 years): 47.3% [95%CI: 45.4, 49.2] ; Older children (6–11 years): 53.1% [95%CI: 51.6, 54.7]; Adolescents (12–19 years): 54.3 [95%CI: 52.6, 55.9]; Adults (20–64 years): 39.4% [95%CI: 38.7, 40.1]; Elderly (≥ 65 years): 36.3% [95%CI: 35.3, 37.4] p < 0.001							
	Marchese, 2021	Adjusted beta, 95%CI and p value	Gender: (Male = reference) Female: -0.8% [95%CI: -2.2, 0.5], p = 0.308	Age (19-30 = reference): 31–50:–4.6% [-6.4, -2.9]; 51-70: -8.3% [-10.3, -6.4]; 71+: -5.5% [-8.0, -2.9] p <0.00l.	Country of birth (Australia = reference): Main English-speaking country: -1.2% [-2.9, 0.4]; Other: -8.1% [-9.8, -6.3] p <0.001	Area-level disadvantage (First quintile (greater disadvantage) = reference): Second quintile: -1.0% [-2.8, 0.8]; Third quintile: -0.1% [-1.9,1.7]; Fourth quintile: -0.7% [-2.9, 1.4]; Fifth quintile:-2.4% [-4.6, -0.1] p = 0.048	Education (Low = reference): Medium: -0.8% [-2.5, 0.8], High: -2.3% [-4.5, -0.2] p = 0.005	Household income (First quintile (20 % lowest income) = reference): Second quintile: 3.4% [1.7, 5.1]; Third quintile: l.9% [0.2, 3.5]; Fourth quintile: 2.2% [0.3, 4.2]; Fifth quintile (20 % highest income): -1.2 [-3.1, 0.7] p = 0.011	Rural/urbani sation (Major city of Australia = reference) Inner regional Australia: 0.6% [-0.8, 2.1]; Other: 0.3% [-1.8, 2.3] p= 0.904	
**Barbados**	Harris, 2022	Unadjusted mean kcal/d (95 % CI) standardised energy intake to 2000 kcal/d, 95%CI and p value	Age: 25–44: 889.1 kcal/d [95%CI: 835.8, 942.3]; 45–64: 737.1 [692.8, 781.3] p < 0.05	Gender: Males: 802.4 kcal/d [750.8, 854.1]; Females: 811.3 [763.4, 859.2] p > 0.05	Education: <Tertiary: 818.5 kcal/d (778.4, 858.5); Tertiary: 768.3 kcal/d [696.0, 840.7] p > 0.05					
**Belgium**	Vandevijver e, 2018	Unadjusted mean and 95%CI and adjusted beta, SE and p value	Unadjusted Gender (2004, 15-64 year-olds) Females: 28.9% [95%CI: 27.1, 30.2]; Males: 32.3% [30.9, 34.3]; (2014-15) Females: 29.7% [95%CI: 28.7, 31.2]; Males: 29.9% [28.6, 31.2]	Adjusted age (2014-15) (3–5 = reference): 6-9:-6.98% (SE:1.83), p = 0.0001; 10-13: -6.58% (SE:1.79), p = 0.0002; 14-17: - 6.23 (SE:1.80), p = 0.0006; 18-34: - 7.27 (SE:2.10), p = 0.0006; 35-50: - 8.56 (SE:2.11), p < 0.0001; 51-64: 0.47 (SE:2.10), p = 0.8241	Unadjusted Education level (2014-15): Secondary education or lower: 30.5% [95%CI: 28.6, 31.5]; Higher education, short type: 29.9% [95%CI: 28.0, 31.4] Higher education, long type: 30.5% [95%CI:28.9, 31.9]	Adjusted Region (2014-15) (Flanders = reference) Brussels capital region: 6.13% (SE: 1.17) p < 0.0001; Walloon region: 8.09% (SE: 0.78) p < 0.0001				
	Vandevijver e, 2020	Unadjusted mean and 95%CI	Unadjusted Gender Females: 29.7% [95%CI: 28.7, 31.2]; Males: 29.9% [95%CI: 28.6, 31.2]	Unadjusted Age: 3–9 years: 33.3% [95%CI: 32.1, 35.0]; 10–17 years: 29.2% [95%CI: 27.7, 30.3]; 18–64 years: 29.6% [28.5, 30.7]	Unadjusted Education level: Low: 30.5% [95%CI: 28.6, 31.5]; Medium: 29.9% [95%CI: 28.0, 31.4]; High: 30.5% [95%CI: 28.9, 31.9]					
**Brazil**	Verly-Jr, 2021	Unadjusted mean and 95%CI	Unadjusted Income: <0.5MW: 16.7% [95%CI: 16.1, 17.3]; 0.5-1 MW: 22.4% [95%CI: 21.9, 22.9]; 1.5-3MW: 27.3% [95%CI: 26.6%, 28.1%]; >3MW: 31.8 [95%CI: 30.9, 32.8]							
	Louzada, 2017	Unadjusted mean and 95%CI	Household income per capita: Tertile 1 (R$ 149.4–567.2 per capita): 15.7%; Tertile 2 (R$ 567.3–843.5 per capita): 22.4%; Tertile 3 (R$ 843.5–6445.4 per capita): 28.5% p <0.001							
	Louzada, 2015	Unadjusted p-value across quintiles of UPF intake	Age (10-19, 20-39, 40-59, 60+) p < 0.001	Gender (Male, Female) p < 0.001	Race/ethnicity (White, African-descendent, Other) p < 0.001	Rural/urbanisation (Rural, Urban) p < 0.001	Years of education (≤ 4, 5-8, 9-12, >12) p < 0.001	Annual household income per person (USD) (≤2200, 2201-4400, >4400) < 0.001		
	Canella, 2018	Unadjusted mean intakes %kcal/day, 95%Cis	Age: 10–19: 26.8% [95%CI: 26.1– 27.6]; 20–39: 21.3% [20.8, 21.9]; 40–59: 17.2% [16.6, 17.8]; 60+: 15.0% [14.2, 15.8]	Gender: Male: 19.2% [95%CI: 18.7, 19.7]; Female: 21.8% [21.3, 22.2]	Region: North: 14.8% [95%CI: 14.3, 15.4]; Northeast: 14.9% [14.5, 15.3]; Southeast: 23.6% [23.0, 24.2]; South: 25.7% [25.0,26.4]; Midwest: 19.4% [18.4, 20.3]	Rural/urbanisation: Rural: 12.7% [12.3, 13.2]; Male: 22.1% [21.7, 22.5]	Household income per capita: 1st tercile: 15.1% [95%CI: 14.6, 15.5]; 2nd tercile: 20.2% [19.7, 20.8]; 3rd tercile: 26.3% [25.7, 26.9]			
	Nilson, 2022	Unadjusted mean intakes %kcal/day, 95%Cis	Age (stratified by gender Male (M); Female (F)): 15-19: M: 25.1% [95%CI: 23.3, 26.9]; F: 26.2% [24.5, 28.0]; 20-24: M: 22.8% [20.8, 24.9]; F: 25.0% [23.5, 26.5]; 25-29: M: 22.3% [20.8, 23.9]; F: 22.0% [20.7, 23.3]; 30-34: M: 18.4% [17.2, 19.6]; F: 21.0% [19.3, 22.7]; 35–39: 18.6% [16.7, 20.4]; F: 19.0% [17.9, 20.1]; 40–44: M: 15.5% [14.4, 16.6]; F: 18.5% [17.3, 19.7]; 45-49: M: 18.1% [16.4, 19.6]; F: 18.4% [17.0, 19.8]; 50-54: M: 15.4% [14.3, 16.6]; F: 17.4% [15.9, 18.8]; 55-59: M: 14.6% [13.6, 15.7]; F: 16.2% [15.2, 17.3]; 60-64: M: 13.0% [11.9, 14.1]; F: 16.3% [15.2, 17.4]; 65-69: M: 14.2% [13.0, 15.5]; F: 16.0% [14.5, 17.5]; 70-74: M: 14.4% [12.6, 16.2]; F: 16.2% [14,7, 17.7]; 75-79: M: 13.1% [11.4, 14.8]; F: 15.1% [13.4, 16.7]; 80+: M: 12.7% [10.5, 15.0]; F: 17.9% [13.7, 22.1]							
**Canada**	Moubarac, 2016	Unadjusted mean and p value	Gender: Female: 46.5%; Male: 48.6% p < 0.001	Age: 2-18: 55.1%; 19-30: 51%; 31-50: 44.9%; 51-64: 42.2%; 65+: 42.6 p < 0.001	Education level: Less than high school diploma: 51.7%; High school diploma: 47.6%; Postsecondary studies: 49%; Post-secondary studies diploma: 44.1% p < 0.001	Family income: Low: 47.1%; Low-medium: 47.6%; Medium-high: 47.9%; High: 47.7%, p < 0.05	Rural/urbanisation: Rural: 50%; Urban: 47.2% p < 0.001			
	Nardocci, 2018	Adjusted standardised beta and p value	Gender: (Male = reference) Female: -0.04, p = 0.005	Age (continuous): - 0.14, p < 0.001	Education (< Postsecondary graduation = reference): Postsecondary graduation: -0.06, p < 0.001	Income (Lowest = reference) Lowermiddle: 0.02, p = 0.277; Upper-middle: 0.02, p = 0.346; Highest: -0.01, p = 0.850; Not stated: 0.02, p = 0.318	Immigration status (Nonimmigrant = reference): Immigrant: -0.22, p < 0.001	Rural/urbanisation (Rural = reference): Urban: -0.01, p = 0.213		
	Polsky, 2020	Unadjusted mean, 95%CI and p value	Age and gender 2004: 2-5: 51.0% {95%CI: 49.8, 52.3]; 6-12: 55.8% [55.0, 56.6]; Adolescent females 13-18: 57.2% [56.1, 58.3]; Adolescent males 13-18: 57.4% [56.2, 58.5]; Adult females 19-54: 44.8% [43.8, 45.8]; Adult males 19-54: 48.2% [47.0, 49.4]; Older females 55+: 41.7% [40.6, 42.8]; Older males 55+: 42.5% [41.5, 43.6] 2015: 2-5: 48.0% [46.1, 49.9]; 6-12: 53.0% [51.9, 54.2]; Adolescent females 13-18: 50.4% [48.5, 52.4]; Adolescent males 13-18: 53.2% [51.5, 54.9]; Adult females 19-54: 41.6% [40.2, 43.0]; Adult males 19-54: 45.4% [43.8, 47.0]; Older females 55+: 45.2% [44.0, 46.4]; Older males 55+: 45.3% [43.9, 46.7]							
	Hutchinson, 2021	Adjusted mean SE and p value	Food security across age-sex groups (Food secure, Marginally food insecure, Moderately food insecure, Severely food insecure: 1-8-years: p-trend = 0.002; 9-18- years: p-trend = 0.049; Women 19-64-years: p-trend = 0.003; Men 19-64-years: p-trend = 0.009							
	Nardocci, 2020	Unadjusted p-value across tertiles of UPF intake	Age(19-30, 31-50, 51-64, 65+): p = 0.0004	Gender (Male, Female) p = 0.0006	Income (quintiles) p = 0.0143	Education level (< High school, High school, Trade/college/CEGEP, University diploma) p < 0.0001	Rural/urbanisation (Rural, Urban) p < 0.0001	Immigrant (Immigrant, Canadian-born) p < 0.0001	Indigenous identity (Indigenous, non- Indigenous) p = 0.0009	
**Chile**	Cediel, 2017	Adjusted mean, 95%CI and p value	Gender: Female: 29.4% [95%CI: 28.1, 30.6]; Male: 27.8% [26.5, 29.2], p > 0.05	Age: 2-19: 38.6% [95%CI: 36.7, 40.6], 20-49: 26.7% [25.2, 28.2]; 50-64: 21.8% [19.7, 24.0], >=65: 18.3% [16.8, 19.8] p-trend <= 0.001	Rural/urbanisation: Rural: 23.7% [95%CI: 21.9, 25.5]; Urban: 29.3% [28.3, 30.4] p < 0.05	Region of country North: 27.5% [95%CI: 24.4, 30.6] (c); Centre: 28.5% [26.8, 30.3] (c); South: 26.7% [24.8, 28.6] (c,d); South (Austral): 27.3% [24.2, 30.4] (c); Metropolitan: 30.2% [28.6, 31.8] (e) unlike letters (c-e) p < 0.05	Family income: 1x MW: 25.8% [95%CI: 24.0, 27.6]; 2xMW: 28.7% [27.2, 30.3]; 3-5x MW: 30.0% [27.8, 32.2]; >= 6x MW: 30.1% [28.3, 31.9] p-trend <= 0.001	Head of family years of school: <=8 years: 28.7% [95%CI: 27.3, 30.1]; 9-11 years: 27.4% [25.7, 29.1]; >=12 years: 29.8% [28.0, 31.6]		
**Colombia**	Khandpur, 2020	Adjusted mean intake, SE, beta and p value	Gender Female: 16.2% (SE:0.2); Male: 15.5 (0.2); (Female = reference) Male beta: -0.6, p = 0.007	Age: 2-9: 19.3% (SE:0.3); 10-19: 19.3% (0.2); 20-34: 15.4% (0.3); 35-49: 12.2% (0.3); >=50: 11.4% (0.4); (2-9 = reference): 10-19 beta: -0.1, p = 0.718; 20-34 beta: - 3.9, p < 0.001; 35-49 beta: -7.1, p < 0.001; >=50: -7.9, p < 0.001	Socioeconomic status: Level 1 (low): 12.7% (SE: 0.3); Level 2: 15.8% (0.3); Level 3: 17.9% (0.3); Level 4 (high): 22.8% (1.0); (Level 1 (low) = reference): Level 2 beta: 3.1, p < 0.001; Level 3 beta: 5.2, p < 0.001; Level 4 (high) beta: 10.1, p < 0.001	Rural/urbanisation: Urban: 17.3% (SE: 0.2); Central: 12.6% (0.5); Rural: 11.2% (0.6); (Urban = reference): Central beta: -4.6, p < 0.001 Rural beta: -6.1, p < 0.001	Region: Atlantic: 12.7% (SE:0.3); Eastern: 18.1% (0.4); Central: 14.4% (0.4); Pacific: 14.9% (0.4); Bogotá: 21.6% (0.5); Orinoquía and Amazon: 15.7% (0.6); (Atlantic = reference): Eastern beta: 5.4, p < 0.001; Central beta: 1.7, p = 0.001; Pacific beta: 2.2, p < 0.001; Bogotá beta: 8.9, p < 0.001; Orinoquía and Amazon beta: 3.0, p < 0.001			
**France**	Calixto Andrade, 2021	Adjusted mean intake and 95%CI	Gender: Male: 31.4% [95%CI: 30.1, 32.7]; Female: 30.9% [30.0, 31.9]	Age: 18-39: 39.1% [95%CI: 37.8, 40.5] 40-59: 28.1% [27.2, 29.0] 60+: 21.6% [20.4, 22.8]	Rural/urbanisation: Rural: 28.9% [95%CI: 27.4, 30.4]; Urban: 31.9% [95%CI: 30.9, 32.8]	Occupation: Management/interm ediate profession: 32.2% [95%CI: 30.9, 33.4]; Self- employed/farmers: 28.1% [25.1, 31.2]; Manual workers/employees: 32.7% [31.3, 34.2]; Retired: 22.3% [21.1, 23.5]; Homemakers, disabled persons, and others: 35.9% [34.1, 37.7]	Education: Incomplete high school: 26.5% [95%CI: 24.9, 28.1]; Complete high school: 32.9% [31.8, 34.1]; Technical course: 32.2% [30.3, 34.0] University degree: 31.9% [30.4, 33.4]			
	Salomé, 2021	Unadjusted p-value across tertiles of UPF intake	Age (18-24, 25-34, 35-49, 50-65, 65-79) p < 0.001	Gender (Male, Female) p = 0.603	Education (Primary school, Middle school, Secondary school, College or university) p < 0.001	Occupation (Employee, Manual worker, Farmer, Craftsman, shopkeeper, business owner, Intermediate profession, Professional, executive, Retired, Inactive) p < 0.001	Region (Ile-de-France (Paris area), North-West, North-East, South-East, South-West) p = 0.238	City size (Rural, 2 000-19 999 individuals, 20 000-99 999 individuals, ≥100 000 individuals, Paris agglomeration) p < 0.001	Food insecurity (Food security, Moderate food insecurity, Severe food insecurity) p < 0.001	Marital status (Single, Married, Unmarried couple, Widowed, Separated/divorce d, Refusal to answer) p < 0.001
**Italy**	Ruggiero, 2021	Adjusted beta, 95%CI and p value	Gender (Women = reference) Men beta: -1.28 [95%CI: -1.89, - 0.68] p < 0.0001	Age (20-40 = reference) 41-65 beta: -1.15 [95%CI: -2.14, -0.15] p < 0.0001, >65 beta: - 3.10 [-4.40, -1.80] p = 0.024	Geographical area (Northern Italy = reference) Central Italy beta: -0.23 [95%CI: -0.99, 0.53] p = 0.56; Southern Italy = 0.014	Rural/urbanisation (Rural = reference) Urban beta: 1.64 [95%CI: 0.87, 2.42] p < 0.0001	Education (Upper Elementary = reference) Lower secondary beta: 0.70 [95%CI: -0.15, 1.55] p = 0.11; Upper secondary beta: 0.55 [-1.36, 0.74] p = 0.20; Postsecondary beta: 0.65 [-2.14, 0.44] p = 0.22	Occupation (Manual = reference) Non-manual beta: -0.02 [95%CI: - 0.85, 0.81] p = 0.96; Housewife beta: -0.79 [1.86, 0.29] p = 0.15; Retired beta: -1.87 [2.83, -0.91] p = 0.0001; Student beta: 0.69 [1.60, 2.98] = 0.55; Unemployed beta: -0.64 [-2.30, 1.01] p = 0.44	Marital status (Married/in couple = reference) Unmarried beta: 1.26 [95%CI: 0.37, 2.15] p = 0.0053; Separated/divorced beta: 1.88 [0.38, 3.38] p = 0.014; Widowed beta: 1.16 [0.07, 2.24] p = 0.037	
**Korea**	Shim, 2022	Adjusted mean intake, 95%CI and p value	Gender: Female: 25.0% [95%CI: 24.4, 25.6]; Male: 25.8% [95%CI: 25.5, 26.1] p < 0.0001. Time trend across 2010-18 p-trend Males <0.0001; Females <0.0001	Age: 1–12: 30.7% [95%CI: 30.0, 31.3]; 13–19: 33.8% [32.9, 34.6]; 20–49: 26.6% [26.1, 27.0]; 50–64: 19.7% [19.3, 20.1]; ≥65: 16.3% [15.8, 16.7]; p linear trend < 0.001. Time trend across 2010-18 p- trend, 1-12 = 0.0002; 13-19 = 0.0001; 20-49 <0.0001; 50-64 <0.0001; ≥65 <0.0001	Residence: Urban: 25.8% [95%CI: 25.5, 26.1]; Rural: 25.0% [24.4, 25.6] p = 0.004. Time trend across 2010-18 p-trend, urban <0.0001; Rural <0.0001	Education: Middle school or less: 23.4% [95%CI: 23.0, 23.8]; High school: 26.4% [25.9, 26.9]; College or higher: 26.3% [25.8, 26.9]; p< 0.0001. Time trend across 2010-18 p-trend, Middle school or less <0.0001; High school <0.0001; College or higher: <0.0001	Household income: Low (Quartile 1): 25.5% [95%CI: 24.9, 26.1]; Middle (Quartile 2–3): 25.4% [25.0, 25.8]; High (Quartile 4): 25.3% [24.8, 25.7] p = 0.174. Time trend across 2010-18 p- trend, Low (Quartile 1) = 0.0361; Middle (Quartile 2– 3) <0.0001; High (Quartile 4) <0.0001			
	Sung, 2022	Adjusted mean intake, SE and p value	Gender: Female: 26.19% (SE:0.38); Male: 27.55 (0.39) p = 0.0165	Age 19–29: 34.57% (SE:0.82); 30–49: 27.53% (0.42); 50– 64: 20.64% (0.41), p < 0.0001, p linear trend < 0.0001. (19-29 = reference) 30-49 p < 001; 50-64 p < 0.001.	Household income: Lowest: 26.22% (SE:0.94); Lower middle: 27.58 (0.53); Upper middle: 26.64 (0.40); Highest: 26.80% (0.46), p = 0.4254, p linear trend = 0.8007	Education level: Middle school or lower: 24.98% (SE:0.66); High school: 27.59 % (0.43); College or higher: 26.81% (0.37), p = 0.0022, p linear trend = 0.2662. (Middle school or lower = reference) High school p < 0.01; College or higher p < 0.05.	Rural/urbanisation: Urban: 27.01% (SE:0.29); Rural: 25.88% (0.66); p = 0.1138	Marital status: Single/Separated/Divorced: 27.73% (0.62); Married: 26.43% (0.37); p = 0.1169	Household status: One- person household: 27.57% (SE:0.29); Multiperson household: 26.81% (0.91) p = 0.4439	
		Shim, 2021	Unadjusted mean, SE and p value	Gender: Male: 27.1% (SE:0.3); Female 25.3% (0.3), p < 0.001	Age: 1-18: 31.5% (SE:0.4); 19-49: 29.9% (0.3); 50-64: 21.0% (0.3); 65+: 15.8% (0.3), p < 0.001						
	Shim, 2021	Unadjusted p-value across tertiles of UPF intake	Age (continuous) p < 0.001	Gender (Male, Female) p < 0.001	Income (High (above median income), Median and below income)n p = 0.082	Rural/Urbanisation (Urban, Rural) = 0.001	Education (>12 years, <=12 years) p <0.001			
**Mexico**	Marrón-Ponce, 2019	Unadjusted p-value across quintiles of UPF intake	Gender (Male, Female) p = 0.16	Age (1-4, 5-11, 12-19, 20-59, 60+) p < 0.001	Rural/Urbanisation (Rural, Urban) p < 0.001	Region (South, Central, North) p < 0.001	Socioeconomic status (Low, Medium, High) p < 0.001	Head of household educational level (No formal education, Elementary school, Middle school, High school, College) p < 0.001		
		Marrón-Ponce, 2017	Adjusted beta and 95%CI	Gender (Male = reference) Female beta: 0.5% [95%CI: -0.9, 1.9]	Age (Pre-schoolaged children = reference) Schoolaged children beta: -3.8% [95%CI: -5.4, -2.2]; Adolescents beta: -3.0% [-4.9, - 1.1]; Adults beta: - 12.5% [-14.1, -10.9]	Rural/Urbanisation (Rural = reference) Urban beta: 5.6% [95%CI: 4.2, 7.0]	Region of Mexico (South = reference) Central beta: 2.7% [95%CI: 1.2, 4.1]; North beta: 8.4% [6.6, 10.1]	Socioeconomic status (Low = reference) Medium = 4.5% [95%CI: 2.8, 6.2]; High = 4.5% [2.5, 6.5]	Head of household education level (Without education = reference) Elementary education: 1.9% [95%CI: -0.5, 4.3]; Middle school education: 3.4% [0.8, 6.1]; High school education: 4.3% [1.1, 7.4]; College graduate education: 7.8% [4.3, 11.4]		
	Oviedo-Solís, 2022	Unadjusted mean and 95%CI	Age: (Dietary recall) Adults (<60): 21.4% [95%CI: 18.8, 24.0]; Older adults (60+): 14.2% [10.7, 17.6]; (Food Frequency Questionnaire) Adults (<60): 20.9% [95%CI: 18.5, 23.2]; Older adults (60+): 16.6% [13.6, 19.7]							
**Netherlan ds**	Vellinga, 2022	Unadjusted mean (g/2000kcal), 95%CI and p value	Gender: Male: 889g/2000kcal [95%CI: 870, 907]; Female: 898g/2000kcal [877, 918] p > 0.05	Age: 1-3: 1202g/2000kcal [95%CI: 1159, 1246]; 4-8: 1252g/2000kcal [1217, 1288]; 9-13: 1209g/2000kcal [1175, 1243]; 1418: 1165g/2000kcal [1124, 1206]; 1930: 962g/2000kcal [921, 1003]; 31-50: 874g/2000kcal [834, 914]; 51-70: 700g/2000kcal [669, 730]; 71-79: 632g/2000kcal [607, 656] p < 0.001	Education level: Low: 871g/2000kcal [95%CI: 838, 903]; Moderate: 939g/2000kcal [916, 962]; High: 850g/2000kcal [830, 871] p < 0.001	Degree of urbanisation: Low: 876g/2000kcal [856, 896]; Moderate: 898g/2000kcal [868, 928]; High: 916g/2000kcal [891, 942] p < 0.01				
**Portugal**	Miranda, 2020	Unadjusted mean, SE and p value	Age: Adults (18-64): 23.84% (SE: 0.42); Elderly (65+): 15.96% (SE: 0.56), p < 0.001							
	Magalhães, 2021	Adjusted beta and 95%CI	Age by gender (Male (M); Female (F)): (45-64 = reference) 3-9: M: 179g [95%CI: 128, 231]; F: 140g [89, 191]; 10-17: M: 327g [277, 377]; F: 192g [135, 249]; 18-44: M: 235g [190, 280]; F: 100g [67, 133]; 65-84: M: -51g [-93, -9] ; F: -63g [-91, -34]	Region by gender (Male (M); Female (F)): (North = reference): Centre: M: 0g [95%CI: -51, 52]; F: 7g [-26, 40]; Lisbon Metropolitan Area: M: 76g [19, 133]; F: 39g [-3, 81]; Alentejo: M: 41g [23, 106]; F: 50g [9, 90]; Algarve: M: 32g [-17, 80]; F: 36g [1, 70]; Autonomous Region of Madeira: M: -7g [-53, 39]; F: -23g [-59, 13]; Autonomous Region of Azores: M: 82g [-3, 167]; F: 40 [-3, 90]	Education by gender (Male (M); Female (F)): (>12 years = reference): <=6 years: M: -68g [95%CI: -124, -12]; F: -51g [-86, - 16]; 7-12 years: M: 7g [-32, 46]; F: 21g [-6, 49]	Urbanisation by gender (Male (M); Female (F)): (Predominantly urban area = reference): Medially urban area: M: 1g [95%CI: -65, 67]; F: -12g [-49, 24]; Predominantly rural area: M: 0g [-47, 48]; F: -21g [-61, 20]	Civil status by gender (Male (M); Female (F)): (Single, divorced or widowed = reference): Married, couples: M: -48g [95%CI: - 96, -1]; F: -10g [-38, 17]	Household members by gender (Male (M); Female (F)): (1-2 = reference): 3-4: M: 13g [95%CI: -29, 54]; F: -6g [-37, 25]; 5+: M: 7g [-63, 77] ; F: -25g [-79, 29]	Food insecurity by gender (Male (M); Female (F)): (No = reference): Yes: M: -43g [95%CI: - 109, 23]; F: - 11g [-43, 22]	
**Spain**	Romero Ferreiro, 2021	Pearson correlation coefficient and p-value	Age: p = -0.53, p <0.0001							
	Romero Ferreiro, 2022	Adjusted beta, SE and p value, p value across time	Age (continuous) beta: -0.15 (SE:0.01) p < 0.001. Across time (5-24, 25-49, 50-75): DRECE I 1991: p < 0.001; DRECE II 1996: p < 0.001; DRECE III 2004: p = 0.014; DRECE IV 2008: p = 0.035	Gender (Male = reference) Female beta: 1.06 (SE:0.33), p = 0.01. Time trend (Male, Female) DRECE I 1991: p = 0.589; DRECE II 1996: p < 0.001; DRECE III 2004: p = 0.031; DRECE IV 2008: p = 0.401	Geographical region (North-West, North, North-East, West, Central-South, East, South, Canary Islands) DRECE I 1991: p < 0.0001, DRECE II 1996: p = 0.010; DRECE III 2004 p < 0.001; DRECE IV 2008: p < 0.001					
	Blanco-Rojo, 2019	Unadjusted p-value across quartiles of UPF intake	Gender (Male, Female) p trend = 0.39	Age (continuous) p trend < 0.001	Education level (No formal education, Primary, Secondary or higher) p trend < 0.001	Household status (Living alone, Living with others) p trend < 0.001				
**Switzerland**	Bertoni Maluf, 2022	Unadjusted median, IQR and unadjusted and adjusted p-value	Gender: Male: 29.2% [IQR: 20.8– 39.9]; Female: 28.4% [19.4, 38.5] adjusted p = 0.012	Age: 18-29: 34.8% [IQR: 24.5, 45.0] ; 30-39: 31.8% [22.3, 42.0]; 40-49: 28.2% [20.3, 37.8]; 50-64: 25.5% [16.9, 36.6]; 65-75: 26.3% [17.1, 35.0] adjusted p = 0.001	Region: German-speaking: 29.6% [IQR: 20.9, 39.6]; Frenchspeaking: 27.2% [17.7, 37.1]; Italianspeaking: 28.0% [16.9, 39.4] adjusted p = 0.002	Nationality: Swiss: 29.2% [IQR: 20.3, 39.0]; Non-Swiss: 26.1% [17.5, 37.1] adjusted p = 0.002	Household status: One person: 29.0% [IQR: 18.5, 40.6]; Two people: 28.1% [19.7, 37.3]; Three people: 28.8% [19.5, 39.7]; Four people and more: 30.2% [21.5, 40.1] adjusted p = 0.400	Education level: Primary & secondary: 29.1% [20.2, 39.7]; Tertiary: 28.4% [19.6, 38.4] adjusted p = 0.060		
**UK**	Lam, 2017	Adjusted beta and 95%CI	Gender (Male, Female): 1.31% [95%CI: -0.99, 3.62]	Age (continuous): - 0.16% [95%CI: - 0.24, -0.09]	Household status: Other adults in household: 0.45% [95%CI: -2.07 to 2.97]; Children in household: 0.54% [-2.18, 3.26]	National Statistics Socio-Economic Classification (NS- SEC): Intermediate vs Managerial & professional: -1.05% ([95%CI: -4.11, 2.02]; Routine & manual vs Managerial & professional: 1.52% [95%CI: -1.02, 4.07]				
	Madruga, 2022	Adjusted trends over time, p value, p for interaction between linear UPF intake trend and sociodemographic characteristic	Gender: Male: p = 0.393; Female: p = 0.983; p for interaction = 0.413	Age: 1-3: p = 0.639; 4-10: p = 0.948; 11-18: p = 0.780; 19-64: p = 0.805 65+: p = 0.278. p for interaction = 0.767	Region: England North: p = 0.258; England Central/Midlands: p = 0.705; England South (including London): p = 0.687; Scotland: p = 0.732; Wales: p = 0.880; Northern Ireland: p = 0.218. p for interaction = 0.645	Occupational Social Class: Routine & manual occupations: p = 0.650; Intermediate occupations: p = 0.481; Higher and lower managerial & professional occupations: p = 0.741. p for interaction = 0.740	Race/ethnicity: White: p = 0.559; Mixed ethnic group: p = 0.691; Black or Black British: p = 0.965; Asian or Asian British: p = 0.322; Other race: 0.803. p for interaction = 0.696			
	Adams, 2015	Adjusted mean intake, beta and 95%Cis	Gender (Male = reference) Female beta: -1.38 [95%CI: -2.67 to -0.09]	Occupational Social Class (Managerial & professional = reference) Intermediate beta: 0.34% [95%CI: -1.12, 1.79]; Routine & manual beta: 1.60% [95%CI: -0.05, 3.26]	Age: (Continuous) beta: -0.18% [95%CI: -0.21, -0.14]					
	Rauber, 2019	Unadjusted mean, SE and p value	Age: 1.5-10: 63.53% (SE:0.34); 11-18: 68.00% (0.4); 19-64: 54.89% (0.35); 65+: 52.98% (0.52); (1.5-10 = reference) all age groups p < 0.001							
	Rauber, 2020	Unadjusted mean, SE and p value	Gender: Male: 55.9% (SE:0.6); Female: 52.8% (0.4); p < 0.05	Age: 19-29: 59.2% (1.3); 30-59: 54% (0.4); 60+: 51.8% (0.5); p trend < 0.05	Ethnicity: White: 55.4% (SE:0.4); Non-white: 45.4% (1.2); p < 0.05	Region: England North: 56.1% (SE:0.7); England Central/Midlands: 56.6% (1.0); England South (including London): 51.7% (0.6); Scotland: 56.5% (1.1); Wales: 55.0% (1.0); Northern Ireland: 58.7% (0.8); (England North = reference) England South (including London) p < 0.05; Northern Ireland p < 0.05	Social Class Occupation: Routine & manual: 57.3% (SE:0.7); Intermediate: 53.4% (0.8); Lower managerial & professional: 53.8% (0.7); Higher managerial & professional: 50.3% (0.8); linear p-trend < 0.05			
	Nascimento, 2021	Unadjusted mean and 95%CI	Age: 4-10: 65.7% [95%CI: 64.2, 67.1]; 11-18: 67.1% [65.7, 68.5]; 19+: 54.0% [53.0, 55.0]							
**US**	Kim, 2019	Unadjusted p-value across quintiles of UPF intake	Age (continuous) p < 0.001	Gender (Male, Female) p < 0.001	Race/ethnicity (Non-Hispanic white, Non-Hispanic black, Mexican American, Other) p < 0.001	Poverty level (<130%, 130-<350%, ≥350%) p < 0.001	Education level (Less than high school, High school, More than high school) p < 0.001			
	Juul, 2021	Adjusted trends over time (2001-2 to 2017-18), p-trend values adjusted for multiple comparisons by calculation of false discovery rate q values, and p for interaction between linear UPF intake trend and sociodemographic characteristic	Gender over time: Male: p-trend = 0.001; Female: p-trend = 0.002; p for interaction = 0.06	Age over time: 20-39: p-trend = 0.015; 40-59: p-trend = 0.001; 60+: p-trend = 0.001; p for interaction = 0.15	Ethnicity over time: Non-Hispanic white: p-trend = 0.001, Non-Hispanic black: p-trend = 0.001; Hispanic: p-trend = 0.081; p for interaction = 0.31	Education over time: High school degree: p-trend = 0.001, High school graduate: p- trend = 0.013; Some college: p-trend = 0.001; College graduate: p-trend = 0.049; p for interaction = 0.24	Income over time: <130%: p- trend = 0.024, 130-<350%: p-trend = 0.001, ≥350%: p- trend = 0.001; p for interaction = 0.26			
	Juul, 2018	Unadjusted p-value across quintiles of UPF intake	Gender (Male, Female) p = 0.009	Age (continuous) p < 0.001	Race/Ethnicity (Non-Hispanic white, Non-Hispanic black, Hispanic, Other) p < 0.001	Education level (<9th grade, 9th-11th grade, High school graduate/GED, Some college, College graduate or higher) p < 0.001	Marital status (Married, Separated/divorced/widowe d, Not married) p < 0.001	Family income/poverty ratio (<130%, 130- <350%, ≥350%) p < 0.001		
	Baraldi, 2018	Adjusted mean intake and 95%CI, and p value for linear time trend	Gender: Male: 58.3% [95%CI: 57.6, 59.0]; Female: 58.8% [58.1, 59.5]; p linear trend across time: Male = 0.0368; Female = 0.1834	Age: 2–9: 65.9% [95%CI: 65.0, 66.8]; 10-19: 66.8% [65.9, 67.7]; 20-39: 59.5% [58.7, 60.3]; 40-59: 55.2% [54.1, 56.4]; 60+: 52.8% [51.9, 53.7]; p linear trend <0.05; p linear trend across time: 2-9 = 0.4518; 10-19 = 0.0128; 20-39 = 0.3529; 40-59 = 0.3821; 60+ = 0.1800	Education: Less than high school: 59.55 [95%CI: 58.4, 60.6]; High school: 59.7% [59.1, 60.3]; College: 55.9% [54.6, 57.2]; p linear trend < 0.05; p linear trend across time: Less than high school = 0.1632; High school = 0.0122; College = 0.4667	Family income/poverty ratio: ≤1.30: 59.6% [95%CI: 58.6, 60.7]; 1.31–3.50: 58.7% [57.8, 59.7]; >3.50: 57.7% [56.9, 58.6]; p linear trend < 0.05; p linear trend across time: ≤1.30 = 0.1910; 1.31-3.50 = 0.0380; >3.50 = 0.2310	Race/ethnicity: Non-Hispanic white: 60.2% [95%CI: 59.4, 60.9]; Non-Hispanic black: 60.6% [59.7, 61.5]; 54.8% [53.2, 56.3]; Mexican-American: 54.8% [53.2, 56.3]; Other Hispanic: 52.0% [50.3, 53.7]; Other: 49.6% [47.3, 51.8]; p linear trend < 0.05; p linear trend across time: Non-Hispanic white = 0.0749; Non-Hispanic black = 0.1512; Mexican-American = 0.0501; Other Hispanic = 0.2563; Other Race = 0.4002			
	Steele, 2022	Unadjusted mean, SE and p value	Gender: Male: 55.9% (SE:0.6); Female: 55.0% (0.5); p = 0.123	Age: 20-39: 58.9% (SE:0.6) (a); 40-59: 54.6% (0.8) (b); 60+: 52.2% (0.6) (c); p for trend < 0.001 (unlike letters (a-c) are significantly different p < 0.05)	Race/ethnicity: Mexican American: 53.6% (SE:0.5) (c); Other Hispanic: 47.6% (1.0) (a); Non-Hispanic white: 57.2% (0.5) (b); Non-Hispanic black: 57.3% (0.8) (b); Other race (including multiracial): 45.1% (1.4) (a); p < 0.001; (unlike letters (a-c) are significantly different p < 0.05)	Income:poverty ratio: <1.30: 57.9% (SE:0.7) (a); >1.30–3.50: 56.9% (0.7) (a); >3.50: 53.3% (0.6) (b); Missing: 52.5% (1.3) (b); p < 0.001; (unlike letters (a-b) are significantly different p < 0.05)	Education level: <12 years: 55.9% (0.9) (a); 12 years: 59.6% (0.8) (b); >12 years: 54.0% (0.5) (a); p for trend < 0.001; (unlike letters (a-b) are significantly different p < 0.05)			
	Yang, 2020	Unadjuated median intake, IQR and p value	Gender: Male: 55.0% [IQR: 48.4, 61.7]; Female: 54.8% [47.8, 61.4]; p = 0.325							
	Steele, 2020	Unadjusted mean, SE and p value	Gender: Male: 58.4% (SE:0.4); Female: 58.2% (0.5); p > 0.05	Age: 6-11: 68.2% (SE:0.5); 12-19: 66.9% (0.7); 20+: 55.9% (0.4); linear p-trend < 0.05	Race/ethnicity: Mexican American: 56.8% (SE:0.5) (a); Other Hispanic: 53.5% (0.9) (b); Non-Hispanic white: 59.6% (0.5) (c); Non-Hispanic black: 61.4% (0.8) (c); Other race (including multiracial): 48.6% (1.0) (d); p < 0.001; (unlike letters (a-d) are significantly different p < 0.05)	Family income:poverty ratio: <1.30: 60.5% (SE:0.7) (c); >1.30–3.50: 59.5% (0.7) (bc); >3.50: 56.3% (0.6) (a); Missing: 56.2% (1.2) (ab); p < 0.001; (unlike letters (a-c) are significantly different p < 0.05)				
	Zheng, 2020	Unadjusted p-value across quartiles of UPF intake	Gender (Male, Female) p = 0.004	Age (20-44, 45-59, 60+) p < 0.001	Race/ethnicity (Hispanic, Non-Hispanic White, Non-Hispanic Black, Non-Hispanic Asian, Other races) p < 0.001	Marital status (Married/Living with partner, Widowed//Divorced/ Separated/Never married) p < 0.001	Education level (< High school, High school, > High school) p < 0.001	Annual family income (< $20000, $20000 to < $45000, $45000 to < $75000, ≥ $75000) p = 0.001		
	Steele, 2020	Unadjusted mean, 95%CI and p value; Adjusted mean, 95%CI and p value (place of birth)	Gender: Male: 55.3 [95%CI: 54.5, 56.2]; Female: 56.2% [55.3, 57.0]	Age: 20-39: 58.1% [95%CI: 57.1, 59.0]; 40-59: 54.9% [53.7, 56.1]; 60+: 53.9% [52.8, 55.0] p for linear trend < 0.001	Family income:poverty ratio: <1.30: 56.9% [95%CI: 55.6, 58.1]; >1.30– 3.50: 56.8% [55.8, 57.9]; >3.50: 54.5% [53.6, 55.4]; Missing: 54.4% [52.3, 56.5], p < 0.001	Education level: <12 years: 55.6% [95%CI: 54.1, 57.0]; 12 years: 58.5% [57.1, 60.0]; >12 years: 54.9% [54.1, 55.8]; p for linear trend = 0.023	Race/ethnicity: Mexican American: 54.0% [95%CI: 53.0, 55.0]; Other Hispanic: 49.1% [47.3, 50.9]; Non-Hispanic White: 57.4% [56.4, 58.3]; Non-Hispanic Black: 59.4% [58.0, 60.8]; Non-Hispanic Asian: 38.3% [36.9, 39.7]; Other race (including multi-racial): 57.5% [54.4, 60.5]	Place of birth (adjusted): US-born: 57.9% [95%CI: 57.3, 58.5]; p < 0.001; Foreign-born: 45.4% [44.0, 46.8] p < 0.001		
	Pachipala, 2022	Unadjusted mean, 95%CI and p value	Race/ethnicity: Non-Hispanic Asian American: 39.3% [95%CI: 38.1, 40.5]; Non-Hispanic White: 57.7% [56.9, 58.5]; Non-Hispanic Black: 60.1% [58.8, 61.3]; Hispanic: 52.7% [51.7, 53.6]; Non-Hispanic Other: 57.7% [55.8, 59.6]; (Non-Hispanic Asian American = reference) all p < 0.01	Gender within ethnicity (Male, Female): (Non-Hispanic Asian American = reference) all p < 0.01	Age within ethnicity (18-24, 25-44, 45-64, ≥65): (Non-Hispanic Asian American = reference) all p < 0.01	Marital status within ethnicity (Married, Separated/divorced/ widowed/Not married): (Non-Hispanic Asian American = reference) all p < 0.01	Education level within ethnicity (<High school, High school graduate/General Equivalency Diploma, Some college, ≥College graduate): (Non-Hispanic Asian American = reference) all p < 0.01	Family income:poverty ratio within ethnicity (<1.30, 1.30-3.49, ≥3.50): (Non-Hispanic Asian American = reference) all p < 0.01		
	Buckley, 2019	Unadjusted p-value across quartiles of UPF intake	Gender (Male, Female) p = 0.06	Age group (6-12, 12-19, 20+) p < 0.001; Age (continuous), p < 0.001	Race/ethnicity (Non-Hispanic white, Non-Hispanic black, Mexican American, Asian American, Other) p < 0.001	Family income:poverty ratio (<1.30, 1.30-3.49, ≥3.50): p = 0.007				
	Kim, 2019	Unadjusted p-value across quartiles of UPF intake	Gender (Male, Female) p = 0.79	Age group (6-12, 12-19, 20+) p < 0.001; Age (continuous), p < 0.001	Race/ethnicity (Non-Hispanic white, Non-Hispanic black, Hispanic, Asian American, Other) p < 0.001	Family income:poverty ratio (<1.30, 1.30-3.49, ≥3.50): p = 0.24				
**Multinati onal across Europe (22 countries)**	Mertens, 2021	Unadjusted mean intake and p value	Gender (Male (M); Female (F); p value): Austria: M:31.7%; F: 27.6%; 0.551; Belgium: M: 31.9%; F: 30.2%; 0.972; Croatia: M: 18.5%; F: 19.7%; 0.539; Cyprus: M: 20.3%; F: 21.4%; 0.826; Czech Republic: M: 27.0%; F: 28.2%; 0.619; Denmark: M: 25.3%; F: 24.8%; 0.654; Estonia: M: 17.4%; F: 18.4%; 0.467; Finland: M: 31.0%; F: 32.5%; 0.565; France: M: 28.4%; F: 29.1%; 0.588; Germany: M: 38.0%; F: 38.9%; 0.393; Greece: M: 20.1%; F: 23.7%; 0.311; Hungary: M: 18.0%; F: 17.1%; 0.581; Ireland: M: 31.8%; F: 35.3%; 0.121; Italy: M: 13.0%; F: 13.8%; 0.447; Latvia: M: 32.0%; F: 30.7%; 0.488; The Netherlands: M: 37.0%; F: 37.3%; 0.834; Portugal: M: 19.8%; F: 24.5%; <0.01; Romania: M: 14.6%; F: 15.9%; 0.403; Slovenia: M: 21.7%; F: 23.5%; 0.549; Spain: M: 25.0%; F: 25.3%; 0.947; Sweden: M: 40.6%; F: 43.8%; 0.227; United Kingdom: M: 39.7%; F: 41.3%; 0.369							

**Table 3:** Country-level summary associations between each sociodemographic predictor and UPF intake.

Country	Age	Gender	Race/ethnic ity	Income / income/poverty level	Education level	Socioeconomi c status / Occupation	Food insecurity	Marital status	Household status	Rural/urbanisation	Region of country	Immigrant status / Country of birth	Indigenous identity
**Australia 2011/12**	Unadjusted: younger age. Adjusted: Younger age	Unadjusted: Male or gender not significant Adjusted: gender not significant		Unadjusted: second/third/fourt h household income quintiles. Adjusted: second third and fourth income quintiles (vs. first (lowest) quintile).	Unadjusted: lower education level/less likely to be higher educated. Adjusted: lower education level	Unadjusted: greater arealevel disadvantage/l ower socioeconomic index for area (SEIFA). Adjusted: greater arealevel disadvantage/l ower socioeconomic index for area (SEIFA)				Unadjusted: living in inner regional Australia (lower intake in major cities). Adjusted: rural/urbanisation not significant		Unadjusted: Australian born, or Australian born or from an English country. Adjusted: Australian- or English-speaking-country-born	
**Barbados 2012-13**	Younger age	Gender not significant			Education not significant								
**Belgium 2004 and 2014-15**	2014-15 Unadjusted: Younger age. Adjusted: youngest (3-5) and oldest (51-64) age	2004 Unadjusted: Males. 2014-15 Unadjusted: gender not significant			2014-15 Unadjusted and adjusted: education not significant						2014:15 Adjusted: living in Brussels capital region or Walloon vs. Flanders region.		
**Brazil 2008-9, and 2017-18**	Younger age	2008-9: Female	2008-9: White ethnicity (vs. African- descendent or other)	2008-9: Higher income	2008-9: Higher education level					2008-9: Urban living	2008-9: South and South East regions of Brazil		
**Canada 2004 and 2015**	2004 Unadjusted and Adjusted: Younger age. 2015 Unadjusted: Younger age. From 2004 to 2015, decrease in age-sex groups: 2-5, 6-12, adolescent females and males 1318, adult females and males 1954. Increase in older males and females 55+. Within each food insecurity status: Food secure: highest UPF in adolescents, Very food insecure: UPF high across all ages	2004 Unadjusted and adjusted: Male. 2015 Unadjusted: Males. Within each food insecurity status: no significant difference between adult gender.		2004: Unadjusted and adjusted: Family income (per capita) not significant. 2015 Unadjusted: Income	2004: Unadjusted and adjusted: Lower educaton level. 2015 Unadjusted: lower education level		2015 Adjusted: Higher food insecurity.			2004 Unadjusted: Rural living. Adjusted: rural/urbanisation not significant. 2015 unadjusted: Rural living		2004 Unadjusted and adjusted: Non-immigrant. 2015 Unadjusted: Non-immigrant	2015 Unadjuste d: Indigenou s identity
**Colombia 2005**	Unadjusted and adjusted: younger age	Unadjusted and adjusted: Female				Unadjusted and adjusted: higher SES				Unadjusted and adjusted: Urban living	Unadjusted and adjusted: Bogota region.		
**Chile 2011-12**	Unadjusted and adjusted: younger age	Unadjusted and adjusted: gender not significant		Unadjusted and adjusted: higher family income.	Unadjusted and adjusted: head of household education level not significant					Unadjusted and adjusted: Urban living	Unadjusted and adjusted: Metropolitan region		
**France 2006-7 and 2014-15**	2006-7 and 2014-15 Unadjusted: Younger age	2006-7 and 2014-15 Unadjusted: Gender not significant			2006-7 Unadjusted: Complete high school or above (incomplete high school with the lowest UPF intake). 2014-15 Unadjusted: Middle school more likely to have higher UPF intake, primary school education level more likely to have lower UPF intake	2006-7 and 2014-15 Unadjusted: Not retired (retired with the lowest UPF intake): in a job (employee, manual worker, intermediate profession)	2014-15 Unadjusted: Higher food insecurity	2014-15 Unadjusted: Single/unmarried couple		2006-7 Unadjusted: Urban living. 2014-15 Unadjusted: Living in a city with ≥100 000 habitants with higher UPF intake, rural living more likely to have lower UPF intake	2014-15 Unadjusted: Region of france not significant (Paris, Northeast, Northwest, Southeast, Southwest)		
**Italy 2010-13**	Adjusted: Younger age	Adjusted: Female			Adjusted: Education not significant	Adjusted: Occupation (retired with lower UPF intake vs. manual and non-manual)		Adjusted: unmarried, separated/div orced or widowed (vs. being married/in a couple)		Adjusted: Urban living	Adjusted: North Italy (vs. South)		
**Korea 2010-2018 & 2016-18**	Unadjusted and adjusted: Younger age	Unadjusted and adjusted: Male		2010-18: Unadjusted: Low income. 2010-18 & 2016-18. Unadjusted and adjusted: income not significant	2010-18 Unadjusted and adjusted: mid/high education level (high school or higher). 2016-18 Unadjusted: Higher education level (>12 years of education vs. <= 12 years) Unadjusted and adjusted: mid- high education level (high school or higher)			2016-18 Unadjusted: single/separated/divorced. Adjusted: marital status not significant	Unadjusted: single-person household (vs. multi-person household). Adjusted: household status not significant	2010-18 Unadjusted and adjusted: urban living. 2016-18 Unadjusted: urban living. Adjusted: urban/rural not significant			
**Mexico 2012**	Unadjusted and adjusted: Younger age	Unadjusted and adjusted: Gender not significant			Unadjusted and adjusted: Higher head of household education level (middle school and above)	Unadjusted: Higher SES (lower SES less likely to have high UPF intake). Adjusted: mid-high SES (vs. low SES)				Unadjusted and adjusted: Urban living (>=2500 inhabitants)	Unadjusted: North mexico (South Mexico less likely to have a high UPF intake). Adjusted: North mexico (then Central, lowest in South Mexico)		
**Netherlands 2012-16**	Younger age	Gender not significant			Moderate education level (vs. low or high)					Higher degree of urbanisation			
**Portugal 2015-16**	Unadjusted: Adults (vs. elderly). By gender unadjusted: Younger age (highest in 10-17-year-olds). Adjusted: younger age (highest in 10-17-year-olds).				Unadjusted and adjusted: Higher years of education		By gender unadjusted and adjusted: food insecurity not significant.	By gender unadjusted: single/divorced/widowed (vs. married/couples). Adjusted: female marital status not significant, single/divorced/widowed males (vs married/couples males)	By gender unadjusted: 34 or >5 household members (vs. 1-2), Adjusted: Household status not significant	By gender unadjusted and adjusted: rural/urbanisation not significant	By gender unadjusted: Lisbon Metropolitan area, Azores region. Adjusted: Lisbon Metropolitan area (males), Alentejo and Algarve region (females)		
**Spain 1991, 1991-2008 & 2008-10**	1991 Unadjusted: Younger age. 1991-2008 Adjusted: Younger age. 2008-10 Unadjusted: younger age	1991-2008 Adjusted: Female. 2008-10 Unadjusted: Gender not significant.			2008-10 Undjusted: No formal education less likely to have high UPF intake, primary education likely to have higher UPF intake (similar proportions of secondary or higher education across quartiles of UPF intake)				2008-10 Unadjusted: Living with people (vs. living alone)		Unadjusted: Higher UPF intake in the Canary islands (1991, 1996, 2004, 2008), Northern region (1996, 2004, 2008), Northwest (2004, 2008), West (2004, 2008), lower UPF intake in the East (1991, 1996, 2004), South and Central South (2008).		
**Switzerland 2014-15**	Unadjusted and adjusted: Younger age	Unadjusted: gender not significant. Adjusted: Male			Unadjusted and adjusted: Education not significant				Unadjusted and adjusted: Household size not significant		Unadjusted and adjusted: German-speaking region (vs. Italian- and French-speaking region)	Unadjusted and adjusted: Swiss national (vs. non-Swiss national)	
**UK 2008-9, 2008-12, 2008-14, 2008-16, 2014-16**	Crude and adjusted: Younger age. 2008-16 Adjusted trends: no significant linear trend.	2008-9 Adjusted: Gender not significant. 2008-12 Adjusted: Male. 2008-16: Male. 2008-16 Adjusted trends: no significant linear trend.	2008-16 Unadjusted: White ethnicity (vs. non-white). 2008-16 Adjusted trends: no significant linear trend.			2008-9 Adjusted: Occupational social class not significant. 2008-12 Adjusted: Occupational social class not significant. 2008-16 Unadjusted: lower occupational social class. 2008-16 Adjusted trends: no significant linear trend.			2008-9 Adjusted: Adults or children in household not significant. 2008-16 Adjusted trends: no significant linear trend.		2008-16 Unadjusted: Northern Ireland (lowest UPF intake in South England (including London)). 200816 Adjusted trends: no significant linear trend.		
**US 1988, 2001-18, 2005-14, 2007-12, 2009-14, 2009-16, 2011-16, 2011-18, 2013-14**	Unadjusted and adjusted: Younger age. 2017-18: Adjusted: Oldest adults had highest intake of UPF in 2017-18 (57.4%), but lowest in 2001-2 (51.7%).	1988 Unadjusted: Male. 2005-14 Unadjusted: Female. 2007-12, 2009-16, 2011-16, 2011-18, 2013-14 Unadjusted and adjusted: gender not significant (gender significant in one study from 2011-16). 2001-18 Adjusted trends: increase in males and females over time.	1988 Non-Hispanic white 2001-2018 unadjusted and adjusted: Non-Hispanic black or white, 2011-18: Non-Hispanic black or other (other race with lowest UPF intake in studies from 2005-14 and 2009-16)	1988 Unadjusted: Middle income/poverty ratio (1.3 to <3.5x poverty level), less likely to have higher UPF intake with a higher income/poverty ratio). 2005-18: Lower family income/poverty ratio (either below 1.3x or below 3.5x), or lower annual family income (income not significant in 2013-14). 2001-18 Adjusted trends: increase in UPF intake across all income levels. 2011-18 by ethnicity unadjusted: higher income/poverty ratio (non-Hispanic Asian American), lower income/poverty ratio (less than 3.5x: non-Hispanic White) income/poverty ratio not significant in Hispanic, Non-Hispanic Other and non-Hispanic Black.	1988 Unadjusted: Less likely to have high UPF intake with a less than a high school education. 2001-18: Unadjusted and adjusted: mid education level or mid-low education level. 2011-18 by ethnicity unadjusted: lower education level (non-Hispanic White and non-Hispanic Other), mid-low education level (non-Hispanic Black), higher education level (non-Hispanic Asian American), education level not significant in Hispanics.			2005-14 Adjusted: Marital status (less likely to have a high UPF intake if married). 2011-16 Unadjusted: Widowed, divorced, separated or never married (vs. married/living with a partner). 2011-18 by ethnicity Unadjusted: Not married (non-Hispanic Asian American, non-Hispanic Black and Hispanic), marital status not significant in non-Hispanic White or non-Hispanic Other.				2011-16 Adjusted: US- born (vs. foreign born, true across genders, age groups, income levels, education levels and race/ethnicities).	
**Multination al across Europe**		Unadjusted: Not significant in 21 countries, higher in Portuguese females											

## Data Availability

Further details on the review process and materials are in the supplementary materials. Additional details can be made available upon request.
