# Supplementary material for: Who consumes ultra-processed food? A systematic review of sociodemographic determinants of ultra-processed food consumption from nationally representative samples
Source: Nutr Res Rev. Author manuscript; Available in PMC 2024 Dec 7. (PMC7617046; doi:10.1017/S0954422423000240)
Supplement: Supplementary Material [file EMS189555-supplement-Supplementary_Material.pdf]

**"Who consumes ultra-processed food? A systematic review of sociodemographic determinants of ultra-processed food consumption from nationally representative samples"**

1. **PRISMA checklist**
2. **PRISMA abstract checklist**
3. **Systematic review screening flowchart**
4. **Screening flowchart for inclusion of full texts**
5. **Criterion for awarding Newcastle-Ottawa risk of bias stars**
6. **Newcastle-Ottawa Scale adapted for cross-sectional studies**
7. **Supplementary analyses**

## 1. PRISMA checklist

| Section and Topic | Item # | Checklist item | Location where item is reported |
|---|---|---|---|
| **TITLE** | | | |
| Title | 1 | Identify the report as a systematic review. | Title page 1 |
| **ABSTRACT** | | | |
| Abstract | 2 | See the PRISMA 2020 for Abstracts checklist. | Page 1 |
| **INTRODUCTION** | | | |
| Rationale | 3 | Describe the rationale for the review in the context of existing knowledge. | Page 3 |
| Objectives | 4 | Provide an explicit statement of the objective(s) or question(s) the review addresses. | Page 3 |
| **METHODS** | | | |
| Eligibility criteria | 5 | Specify the inclusion and exclusion criteria for the review and how studies were grouped for the syntheses. | Page 4 |
| Information sources | 6 | Specify all databases, registers, websites, organisations, reference lists and other sources searched or consulted to identify studies. Specify the date when each source was last searched or consulted. | Page 4 |
| Search strategy | 7 | Present the full search strategies for all databases, registers and websites, including any filters and limits used. | Page 4 |
| Selection process | 8 | Specify the methods used to decide whether a study met the inclusion criteria of the review, including how many reviewers screened each record and each report retrieved, whether they worked independently, and if applicable, details of automation tools used in the process. | Page 4 |
| Data collection process | 9 | Specify the methods used to collect data from reports, including how many reviewers collected data from each report, whether they worked independently, any processes for obtaining or confirming data from study investigators, and if applicable, details of automation tools used in the process. | Page 4 |
| Data items | 10a | List and define all outcomes for which data were sought. Specify whether all results that were compatible with each outcome domain in each study were sought (e.g. for all measures, time points, analyses), and if not, the methods used to decide which results to collect. | Page 4 |
| | 10b | List and define all other variables for which data were sought (e.g. participant and intervention characteristics, funding sources). Describe any assumptions made about any missing or unclear information. | Page 4 |
| Study risk of bias assessment | 11 | Specify the methods used to assess risk of bias in the included studies, including details of the tool(s) used, how many reviewers assessed each study and whether | Page 5 |

| Section and Topic | Item # | Checklist item | Location where item is reported |
|---|---|---|---|
| | | they worked independently, and if applicable, details of automation tools used in the process. | |
| Effect measures | 12 | Specify for each outcome the effect measure(s) (e.g. risk ratio, mean difference) used in the synthesis or presentation of results. | Page 5 |
| Synthesis methods | 13a | Describe the processes used to decide which studies were eligible for each synthesis (e.g. tabulating the study intervention characteristics and comparing against the planned groups for each synthesis (item #5)). | Page 6 |
| | 13b | Describe any methods required to prepare the data for presentation or synthesis, such as handling of missing summary statistics, or data conversions. | Page 6 |
| | 13c | Describe any methods used to tabulate or visually display results of individual studies and syntheses. | Page 5 |
| | 13d | Describe any methods used to synthesize results and provide a rationale for the choice(s). If meta-analysis was performed, describe the model(s), method(s) to identify the presence and extent of statistical heterogeneity, and software package(s) used. | Page 5 |
| | 13e | Describe any methods used to explore possible causes of heterogeneity among study results (e.g. subgroup analysis, meta-regression). | Page 5 |
| | 13f | Describe any sensitivity analyses conducted to assess robustness of the synthesized results. | Page 5 |
| Reporting bias assessment | 14 | Describe any methods used to assess risk of bias due to missing results in a synthesis (arising from reporting biases). | Page 5 |
| Certainty assessment | 15 | Describe any methods used to assess certainty (or confidence) in the body of evidence for an outcome. | Page 5 |
| **RESULTS** | | | |
| Study selection | 16a | Describe the results of the search and selection process, from the number of records identified in the search to the number of studies included in the review, ideally using a flow diagram. | Figure 1, Page 5 |
| | 16b | Cite studies that might appear to meet the inclusion criteria, but which were excluded, and explain why they were excluded. | Page 6 |
| Study characteristics | 17 | Cite each included study and present its characteristics. | Page 6 |
| Risk of bias in studies | 18 | Present assessments of risk of bias for each included study. | Table 1 |
| Results of individual studies | 19 | For all outcomes, present, for each study: (a) summary statistics for each group (where appropriate) and (b) an effect estimate and its precision (e.g. confidence/credible interval), ideally using structured tables or plots. | Table 1, page 6 |
| Results of syntheses | 20a | For each synthesis, briefly summarise the characteristics and risk of bias among contributing studies. | Page 8-19 |
| | 20b | Present results of all statistical syntheses conducted. If meta-analysis was done, present for each the summary estimate and its precision (e.g. confidence/credible interval) and measures of statistical heterogeneity. If comparing groups, describe the direction of the effect. | Page 8-20 |
| | 20c | Present results of all investigations of possible causes of heterogeneity among study results. | N/A |
| | 20d | Present results of all sensitivity analyses conducted to assess the robustness of the synthesized results. | N/A |
| Reporting biases | 21 | Present assessments of risk of bias due to missing results (arising from reporting biases) for each synthesis assessed. | N/A |
| Certainty of evidence | 22 | Present assessments of certainty (or confidence) in the body of evidence for each outcome assessed. | Page 24/25 |
| **DISCUSSION** | | | |
| Discussion | 23a | Provide a general interpretation of the results in the context of other evidence. | Page 21-23 |
| | 23b | Discuss any limitations of the evidence included in the review. | Page 22-24 |
| | 23c | Discuss any limitations of the review processes used. | Page 22-24 |

| Section and Topic | Item # | Checklist item | Location where item is reported |
|---|---|---|---|
| | 23d | Discuss implications of the results for practice, policy, and future research. | Page 24 |
| **OTHER INFORMATION** | | | |
| Registration and protocol | 24a | Provide registration information for the review, including register name and registration number, or state that the review was not registered. | Page 3 |
| | 24b | Indicate where the review protocol can be accessed, or state that a protocol was not prepared. | Page 3 |
| | 24c | Describe and explain any amendments to information provided at registration or in the protocol. | Page 3 |
| Support | 25 | Describe sources of financial or non-financial support for the review, and the role of the funders or sponsors in the review. | Page 25 |
| Competing interests | 26 | Declare any competing interests of review authors. | Page 25 |
| Availability of data, code and other materials | 27 | Report which of the following are publicly available and where they can be found: template data collection forms; data extracted from included studies; data used for all analyses; analytic code; any other materials used in the review. | Page 25 |

*From:* Page MJ, McKenzie JE, Bossuyt PM, Boutron I, Hoffmann TC, Mulrow CD, et al. The PRISMA 2020 statement: an updated guideline for reporting systematic reviews. BMJ 2021;372:n71. doi: 10.1136/bmj.n71

## 2. PRISMA abstract checklist

| Section and Topic | Item # | Checklist item | Reported (Yes/No) |
|---|---|---|---|
| **TITLE** | | | |
| Title | 1 | Identify the report as a systematic review. | Yes |
| **BACKGROUND** | | | |
| Objectives | 2 | Provide an explicit statement of the main objective(s) or question(s) the review addresses. | Yes |
| **METHODS** | | | |
| Eligibility criteria | 3 | Specify the inclusion and exclusion criteria for the review. | Yes |
| Information sources | 4 | Specify the information sources (e.g. databases, registers) used to identify studies and the date when each was last searched. | Yes |
| Risk of bias | 5 | Specify the methods used to assess risk of bias in the included studies. | Yes |
| Synthesis of results | 6 | Specify the methods used to present and synthesise results. | In paper |
| **RESULTS** | | | |
| Included studies | 7 | Give the total number of included studies and participants and summarise relevant characteristics of studies. | Yes |
| Synthesis of results | 8 | Present results for main outcomes, preferably indicating the number of included studies and participants for each. If meta-analysis was done, report the summary estimate and confidence/credible interval. If comparing groups, indicate the direction of the effect (i.e. which group is favoured). | Yes |
| **DISCUSSION** | | | |
| Limitations of evidence | 9 | Provide a brief summary of the limitations of the evidence included in the review (e.g. study risk of bias, inconsistency and | Yes |

| Section and Topic | Item # | Checklist item | Reported (Yes/No) |
|---|---|---|---|
| | | imprecision). | |
| Interpretation | 10 | Provide a general interpretation of the results and important implications. | Yes |
| **OTHER** | | | |
| Funding | 11 | Specify the primary source of funding for the review. | In paper |
| Registration | 12 | Provide the register name and registration number. | Yes |

*From:* Page MJ, McKenzie JE, Bossuyt PM, Boutron I, Hoffmann TC, Mulrow CD, et al. The PRISMA 2020 statement: an updated guideline for reporting systematic reviews. BMJ 2021;372:n71. doi: 10.1136/bmj.n71

## 3. Systematic review screening flowchart

**Title and abstract screening flowchart for full-text eligibility analysis**

1. Is it in English?
    - Yes, then:
2. Is it a nationally representative cohort of adults?
    - Yes or maybe, then:
3. Is it observational?
    - Yes or maybe, then:
4. Is there some assessment of individual UPF intake?
    - Yes or maybe, then:
5. Designate for full-text screening.

**Title and abstract screening flowchart for exclusion**

1. Not in English?
    - Exclude
2. Not observational? Animal study, RCT, ecological study or review?
    - Exclude
3. Not an observational sample of adults, and looks at children only or a subgroup of adults eg pregnant females, older adults, with disease?
    - Exclude
4. Clearly no assessment of individual UPF intake (e.g based on sales or household consumption)
    - Exclude

If a paper is in English, is observational, clearly not a subgroup, and defines UPF by NOVA at the individual level, and there is any potential report of predictors of UPF intake, then keep for FT screening.

## 4. Screening flowchart for inclusion of full texts

1. Is it in English?
    - Yes, then:

2. Is it an observational study?
   - Yes, then:
3. Is it a nationally representative cohort of adults?
   - Is it explicitly stated in the paper that the cohort is nationally representative of the country of interest? If it is not explicitly stated, are there other papers of the cohort that describe if it is nationally representative?
   - Does the study focus only on a specific subgroup, or have significant subpopulations been excluded? Exclude if so. Understand that and allow for some exclusions that may be made based on the diet assessment, or minor subgroups for the relevant analysis.
   - Do the participant characteristics match that of the country? If clearly no, then exclude.
   - (If relevant) Has there been weighting to match the national representation if a biased sample?
   - The authors state it is not generalisable to the nation from exclusions/sampling, then exclude
   - (If relevant) If there are multiple countries, is the analysis a nationally representative cohort of adults for each country?
   - Yes, then:
4. Is there assessment of individual UPF intake classified by NOVA?
   - Diet assessment through FFQ, 24-hour recall, diet history etc.
   - Is the outcome a measure of total UPF intake (e.g. absolute or relative, servings per day, grams per day, energy per day)?
   - Yes then:
5. Is there statistical assessment of sociodemographics with UPF intake?
   - A regression model or descriptive statistics.
   - Must report on at least one sociodemographic factor such as age, gender, ethnicity, income, deprivation level, food security, education, marital status, urbanisation, residence area/region and the association of lower/higher levels/values with UPF intake
   - Must include statistical values of the association (p values, confidence intervals, beta coefficients), cannot simply state an association.
   - If there was no evidence of statistical assessment, authors were contacted to provide detail.
   - Yes then:
6. Include the paper in the systematic review.

If papers provided nationally representative samples from the same cohort, these are all reported. Some papers report the same cohort, but they may be from different years.

After papers included by SD and SQ, authors met to discuss any disagreements. After agreement, data was independently extracted.

**5. Criterion for awarding Newcastle-Ottawa risk of bias stars:**

| | Star given | Star not given |
|---|---|---|
| **Representativeness of the sample:** | *Nationally representative sampling methodology | |
| **Sample size** | *Sampling methodology suitable to achieve nationally representative sample | |
| **Non-respondents:** | *Table comparing included and excluded analytical sample based on response rate | No comparison of included and excluded analytical sample |
| **Ascertainment of the exposure** | **Validated tool<br>*Self-report of sociodemographic variables | |
| **Comparability** | *Adjustment for one sociodemographic variable | No adjustment for other sociodemographic variables |
| | *Adjustment for another sociodemographic variable | No adjustment for other sociodemographic variables |
| **Outcome** | **Independent diet assessment by dietitian<br>*Food consumption tool (self-report e.g. 24-hour recall, food frequency questionnaire) | |
| | *Statistical analysis with confidence intervals or p values | |

## 6. Newcastle-Ottawa Scale adapted for cross-sectional studies

Selection: (Maximum 5 stars)

1) Representativeness of the sample:
a) Truly representative of the average in the target population. * (all subjects or random sampling)
b) Somewhat representative of the average in the target population. * (non-random sampling) c) Selected group of users.
d) No description of the sampling strategy.

2) Sample size:
a) Justified and satisfactory. *
b) Not justified.

3) Non-respondents:
a) Comparability between respondents and non-respondents characteristics is established, and the
response rate is satisfactory. *

b) The response rate is unsatisfactory, or the comparability between respondents and non-respondents is unsatisfactory.
c) No description of the response rate or the characteristics of the responders and the non-responders.

4) Ascertainment of the exposure (risk factor): a) Validated measurement tool. **
b) Non-validated measurement tool, but the tool is available or described.* c) No description of the measurement tool.

Comparability: (Maximum 2 stars)
1) The subjects in different outcome groups are comparable, based on the study design or analysis. Confounding factors are controlled.
a) The study controls for the most important factor (select one). * b) The study control for any additional factor. *

Outcome: (Maximum 3 stars)
1) Assessment of the outcome:
a) Independent blind assessment. **
b) Record linkage. **
c) Self report. *
d) No description.

2) Statistical test:
a) The statistical test used to analyse the data is clearly described and appropriate, and the measurement of the association is presented, including confidence intervals and the probability level (p value). *
b) The statistical test is not appropriate, not described or incomplete.

This scale has been adapted from the Newcastle-Ottawa Quality Assessment Scale for cohort studies adapted for cross-sectional studies from the systematic review, “Bullying and health related quality of life among adolescents- a systematic review” (Dubey et al., 2022).

## 7. Supplementary analyses

**By adjusted analyses**

Gender (males with higher intake) became non-significant after adjustment in Australia (Marchese et al., 2021). Gender (males with higher intake) became significant after adjustment in UK (Adams & White, 2015), and males became significant with adjustment in Switzerland (Bertoni Maluf et al., 2022). Income became non-significant after adjustment in Korea (2010-18) (Shim et al., 2021). Differences in UPF intake across social class occupations became non-significant after adjustment in the UK (2008-12) (Adams & White, 2015). Marital status became non-significant after adjustment in Korea (2016-18) (Sung et al., 2021) , and in Portugal in females only (Magalhães et al., 2021). Household status became non-significant after adjustment in Korea (2016-18) (Sung et al., 2021), and in Portugal (Magalhães et al.,

2021). Rurality/urbanisation became non-significant after adjustment in Australia (Marchese et al., 2021), Canada (Nardocci et al., 2018), and  Korea (2016-18) (Sung et al., 2021).
